# Effect of the Polarity of Solvents on Periodic Precipitation: Formation of Hierarchical Revert Liesegang Patterns

**DOI:** 10.1021/acs.jpcb.2c05810

**Published:** 2022-10-11

**Authors:** Gábor Holló, Dániel Zámbó, András Deák, Federico Rossi, Raffaele Cucciniello, Pierandrea Lo Nostro, Hideki Nabika, Bilge Baytekin, István Lagzi, Masaki Itatani

**Affiliations:** † ELKH-BME Condensed Matter Research Group, Budapest University of Technology and Economics, Műegyetem rakpart 3, Budapest1111, Hungary; ‡ Institute of Technical Physics and Materials Science, Centre for Energy Research, Konkoly-Thege Miklós út 29-33, H-1121Budapest, Hungary; § Department of Earth, Environmental and Physical Sciences—DEEP Sciences, University of Siena, Pian dei Mantellini 44, 53100Siena, Italy; ∥ Department of Chemistry and Biology “Adolfo Zambelli”, University of Salerno, Viale Via Giovanni Paolo II 132, 84084Fisciano, Salerno, Italy; ⊥ Department of Chemistry ‘Ugo Schiff”, University of Florence, Via della Lastruccia 3, 50019Sesto Fiorentino, Florence, Italy; # Faculty of Science, Yamagata University, 1-4-12, Kojirakawa, Yamagata990-8560, Japan; ∇ Department of Chemistry and UNAM, Bilkent University, 06800Ankara, Turkey; ○ Department of Physics, Institute of Physics, Budapest University of Technology and Economics, Budafoki út 8, Budapest1111, Hungary

## Abstract

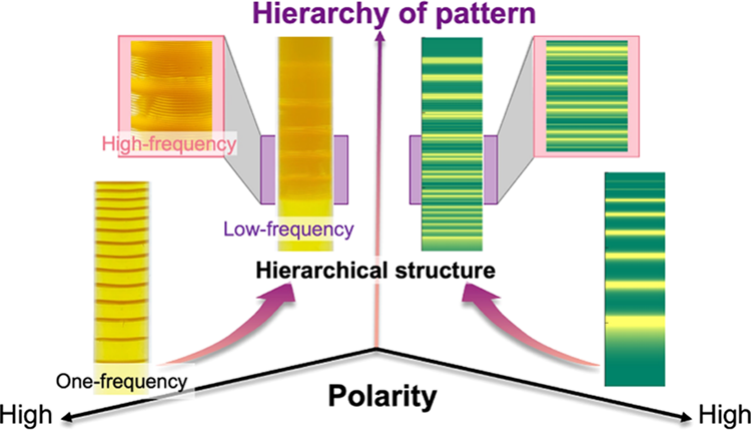

Liesegang pattern (LP) is one example of self-organized periodic precipitation patterns in nonequilibrium systems. Several studies have demonstrated that the LP morphology can track physicochemical environmental conditions (e.g., temperature); however, the polarity effect has not been explored to date. In this study, a copper chromate system is used to reveal the impact of solvent polarity on the evolving LP structure using water/organic solvent mixtures. In the typical case of using water/dimethyl sulfoxide (DMSO) mixtures, two drastic changes in LP morphology with increasing DMSO contents were found: (i) increasing frequency of the original structure and (ii) formation of a hierarchical pattern with the appearance of another, lower-frequency structure. Furthermore, the simulation model operating with a bimodal size distribution, allowing both homogeneous and heterogeneous precipitations showed good agreement with the experimental results. Therefore, this study demonstrated that LP can be tailored by solvent polarity and can be used for designing hierarchical precipitation patterns in a straightforward manner.

## 1 Introduction

Out of equilibrium conditions control various self-assembly and self-organization processes in biological, physical, geochemical, and chemical systems.^1,2^ These processes are important in the formation and design of artificial hierarchical structures with high functionality like those found in nature.^3−5^ The Liesegang phenomenon (Liesegang pattern (LP) or periodic precipitation) is one of the examples of self-organized chemical patterns with periodic layered structures in nonequilibrium systems.^6−8^ This type of pattern formation occurs in a porous medium (typically in hydrogels), in which a homogeneously distributed electrolyte (X^–^, inner electrolyte) is in contact with another medium containing another electrolyte (M^+^, outer electrolyte).^9−11^ A periodic array of precipitate (MX) forms due to the diffusion of M^+^ into the gel and a reaction with X^–^ (M^+^ + X^–^ → MX). The periodicity of the typical LPs shows a general property, namely the distances of bands measured from the gel interface are the members of a geometric progression. This empirical rule is known as the spacing law^12−14^
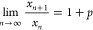
1where *n* is the band number, *x*_*n*_ and *x*_*n*+1_, are the distance of the *n*th and (*n* + 1)th bands measured from the interface between media containing M^+^ and X^–^, and *p* is the spacing coefficient, which is constant at large values of *n*. Furthermore, the inter-band spacing (Δ*x*_*n*_) deduced by the spacing law is given by
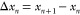
2

Generally, Δ*x*_*n*_ increases with increasing *n*, and this type of pattern is called regular LP.^9,14^ The regular-type LP generation can be observed in the CuCrO_4_ system and many other precipitation systems.^9,14−20^ However, when Pb^2+^ is used instead of Cu^2+^ as an outer electrolyte, exceptional LP can be formed, in which Δ*x*_*n*_ decreases in contrast to the regular-type pattern, which is the so-called revert (invert) LP.^21−23^ This difference was discussed from the perspective of the property of colloidal stability.^24−27^ Also, some LPs sometimes showed the formation of hierarchical layered structures with the coexistence of two patterns with different frequencies of periodicity.^21,28,29^ Furthermore, recent studies on LPs have focused on investigating, in both simulations and experiments, how the periodicity of LPs is controlled and designed in response to physical and chemical conditions of the environment (such as the temperature,^30^ gel concentration,^15^ direct and alternating electric field,^31,32^ and mechanical deformation of the gel^16^). These factors can affect the precipitation by influencing the diffusion of M^+^, the chemical reaction between M^+^ and X^–^, and the nucleation and aggregation of the precipitate.

It is known that solvent polarity affects the stability of colloidal particles.^33−37^ Therefore, the polarity can also modify the routes to precipitation thus affecting the morphology and periodicity of the pattern formed. In this study, we investigate the effect of solvent polarity on the LP structures in the copper chromate precipitation system in an agarose gel. We used a mixture of water and dimethyl sulfoxide (DMSO), a popular aprotic solvent, for the gel preparation, and we changed the volume ratio (φ_DMSO_) to control the polarity of the dispersing media. The relative dielectric constants (ε_r_) of water and DMSO are 78 and 47 at 20 °C,^38^ respectively; since lower ε_r_ indicates a lower polarity, by increasing φ_DMSO_ the polarity in gels is reduced. In our experiments, we found that, by decreasing the polarity of the system, the global structure of the obtained pattern shifted from the regular-type to the revert-type. Furthermore, the regular-type had a fine periodic structure with a length scale (100 μm) smaller than the formed revert pattern itself (∼1 cm). This represents the formation of hierarchical LPs where different periodicities coexist. To extend our concept, we investigated the pattern formation using other common organic solvents (*N*,*N*-dimethylformamide (DMF), ethylene glycol (EG), *tert*-butyl alcohol (TBA), glycerol (GL), and glycerol carbonate (GC)).

## 2 Experimental Section

### 2.1 Reagents and Instrument

Agarose powder (Type I) as a reaction medium, copper (II) chloride (CuCl_2_, 97%) as the outer electrolyte, potassium chromate (K_2_CrO_4_, ≥99.0%) as the inner electrolyte, and EG (≥99.5%), GL (≥99.5%), DMF (≥99%), DMSO (≥99.9%), and TBA (≥99.5%) were purchased from Sigma-Aldrich. All reagents were used without further purification. Pattern formation experiments were carried out in glass test tubes with a diameter of 10 mm. The line profile analysis was performed using ImageJ software. The particle size distribution and ζ potential were measured using dynamic light scattering (DLS) measurement with a Malvern Zetasizer NanoZS (Malvern Panalytical) at 25 °C.

### 2.2 Pattern Formation in Organic Solvents/Water Gels with Different Volume Fractions of Organic Solvents (φ_OS_)

Agarose powder was added to ultrapurified water, and this mixture was continuously stirred using a magnetic stirrer (150 rpm) at 90 °C until complete dissolution of the agarose, making the solution transparent. Subsequently, this solution was cooled at room temperature until the temperature of the solution dropped below 70 °C. This temperature was determined by considering the lowest boiling point of the organic solvents used in this study (TBA, 82 °C). After cooling, the organic solvent was added to this solution with prescribed φ_OS_ between water and organic solvents, and a concentrated aqueous solution of K_2_CrO_4_ (typically 10^–1^ M) was also added to this mixture. This agarose sol was added to a glass test tube at a height of 2:3 from the bottom (usually ∼65 mm). The concentration of agarose and K_2_CrO_4_ were fixed at 1.0% w/v and 1.0 × 10^–2^ M. Subsequently, this sample was cooled in a refrigerator at ∼5 °C overnight to complete the gelation process. After gelation, the gel column was allowed to stand for 1 h at room temperature, and then a mixture of water and organic solvents containing CuCl_2_ (0.5 M, 1.5 mL) was poured on the top of the gel. The same volume ratio of organic solvents/water (φ_OS_) was used in the gel (K_2_CrO_4_) and in the outer electrolyte (CuCl_2_) as well. All experiments on pattern formation were carried out at room temperature, and the time of the pattern formation was 1 week. The periodicity of the patterns was determined using a line profile analysis (Figure S1 in the Supporting information (SI)).

### 2.3 Measurements of the CuCrO_4_ Particle Size

First, the water/organic solvent mixture with K_2_CrO_4_ was prepared in a quartz cuvette (1 cm × 1 cm). Subsequently, the CuCl_2_ solution was added to this mixture and mixed by repeated pipetting up and down 10 times. Concentrations of K_2_CrO_4_ and CuCl_2_ were fixed at 1.0 × 10^–4^ M, only φ_OS_ was changed by adjusting the mixing volume ratio between water and organic solvents. Immediately after mixing, this colloid dispersion was measured by DLS at 25 °C to obtain the size distribution and average particle size.

### 2.4 Measurements of the ζ Potential

The same as in the size measurement, CuCl_2_ was mixed with K_2_CrO_4_ in the water/organic solvent mixture with different φ_OS_. Also, concentrations of CuCl_2_ and K_2_CrO_4_ were the same as in the size measurement. Immediately after mixing, this dispersion was replaced in a folded capillary cell (Malvern DTS1070), then the ζ potential was measured at 25 °C.

## 3 Results and Discussion

**Figure 1:**
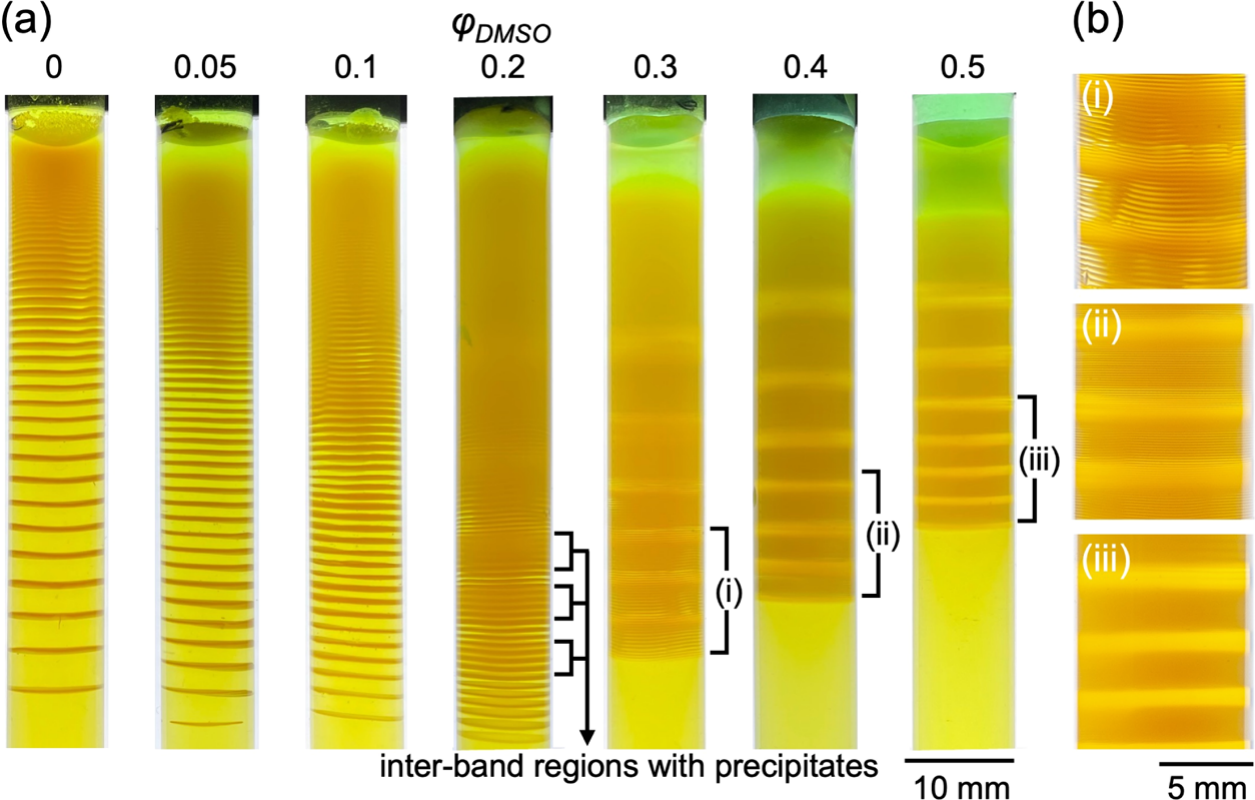
(a) Pattern formation of the CuCrO_4_ system using different volume ratios of DMSO (φ_DMSO_) ranging from φ_DMSO_ = 0 (pure water) to φ_DMSO_ = 0.5 (water:DMSO = 50%:50%). (b) Enlarged optical photographs of regions (i)–(iii) in (a).

Figure 1a shows the patterns obtained after one week at different φ_DMSO_ values. When φ_DMSO_ = 0 (the solvent is pure water), a clear periodic precipitation pattern with alternating precipitation and inter-band (no precipitate) regions was formed. Since Δ*x*_*n*_ increased with the distance measured from the liquid–gel interface, the produced pattern was the regular-type LP. When φ_DMSO_ increased from 0 to 0.1, Δ*x*_*n*_ was found to decrease, thus producing a higher frequency pattern, although the morphology of the regular-type was preserved. In the case of φ_DMSO_ = 0.2, a different trend was observed: while maintaining the overall regular morphology, the formation of a clear precipitate in the inter-band regions was observed. The structure of the pattern was further changed when φ_DMSO_ was increased up to 0.3. The frequency of the bands increased, and the precipitate formed in the inter-band regions self-organized into a secondary structure having a lower frequency with respect to the primary pattern. Interestingly, in the secondary structure, the distance between the precipitate bands became smaller further away from the liquid–gel interface, generating a revert-type LP.^21−23^ However, the primary (higher frequency) structure remained regular and the distance between the bands increased toward the bottom of the test tube (Figure 1b(i)). Therefore, the global periodicity of the pattern switched from regular to the revert-type when φ_DMSO_ was changed from 0.2 to 0.3. High and low-frequency patterns coexisted in the system in a way that the low-frequency pattern is the revert one. Although some previous studies have reported two types of coexistence so far, these are classified into the following two types: coexistence of (i) two independent regular-type structures with high and low frequencies^28,29^ or (ii) revert-type with low frequency and local structure in-between regular- and equidistant-type with high frequency.^21^ Therefore, our pattern structure represents a new class of hierarchical LPs.

On increasing φ_DMSO_ to 0.5, the original high-frequency primary pattern tended to disappear, and it could be seen only in the regions of the low-frequency pattern (Figure 1b(ii),(iii)). Indeed, this change in the distribution of the primary patterns was also observed using optical microscopy (Figure S2 in the SI). Also, the time course of pattern formation for φ_DMSO_ = 0, 0.3, and 0.5 showed that the formation of bands with high-density precipitation and regions with low-density precipitation alternated from top to bottom in both high- and low-frequency patterns (Figures S3–S5 in the SI). Therefore, the phenomena in this system can be explained by a nucleation-particle aggregation-based mechanism (pre-nucleation model) that is frequently employed to understand Liesegang systems.^12,39−41^

To extend the effect of polarity, we also used other organic solvents: DMF (ε_r_ = 40)^42^ as another aprotic solvent and EG (ε_r_ = 38),^43^ TBA (ε_r_ = 12),^44^ GL (ε_r_ = 47), and GC (ε_r_ = 116)^45−47^ as protic solvents (Figures S6–S9 in the SI). In the case of DMF, there was no revert pattern formation, while the frequency of the primary pattern increased as the DMF content increased (Figure S6 in the SI). In contrast, EG and TBA showed a transition similar to the case observed in DMSO (Figures S7 and S8 in the SI). Therefore, the pattern transition from regular to revert-type and formation of the low-frequency pattern structure occurred regardless of the type of solvents (protic or aprotic). Furthermore, it was suggested that periodicity could be controlled by the polarity because the length scale of both structures with lower and higher frequency depending on the type and the volume ratios of the solvents.

**Figure 2:**
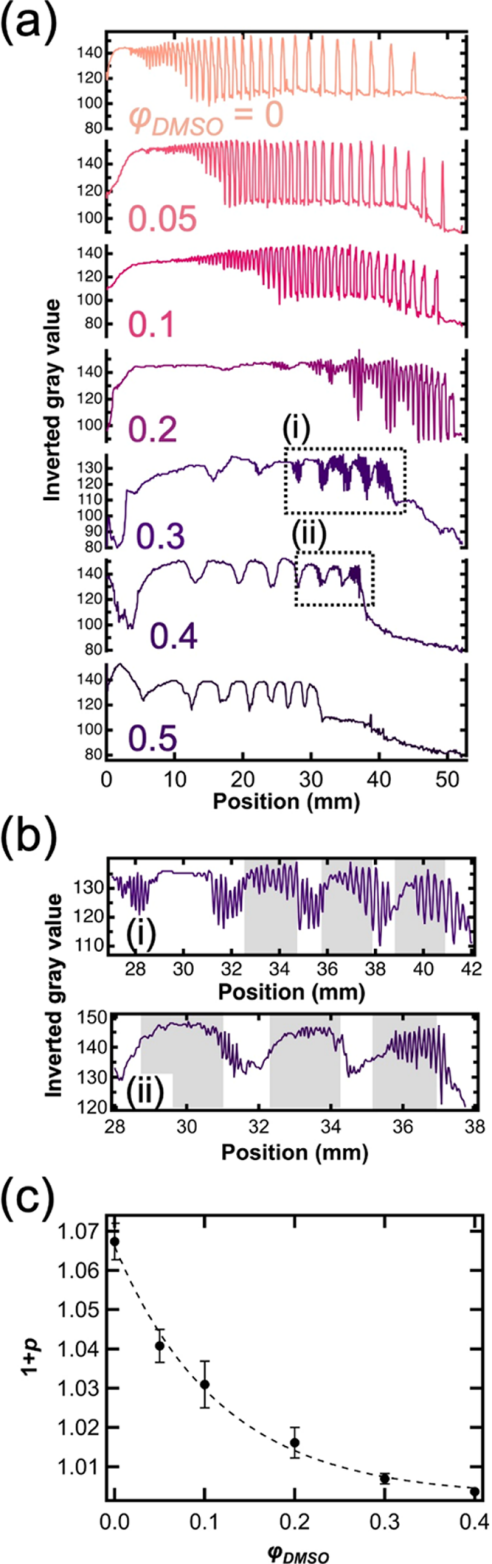
(a) Line profile analysis of the LPs starting from the liquid–gel interface (position = 0) to the bottom of test tubes at various DMSO contents. (b) Enlarged views of the regions (i) and (ii) are indicated as dotted line squares in (a). (c) Relationship between the spacing coefficient (*p*) and φ_DMSO_. The plot was fitted by a power function (*f*(*x*) = *f*(0) + α*x*^β^, where α and β are coefficients). Error bars were obtained from 5 replicates, which were calculated using *p*-values < 0.05.

To explore the details of the abovementioned transition of the morphology and the periodicity of the obtained patterns, a line profile analysis in Figure 1a was performed (Figure 2a). When φ_DMSO_ = 0–0.1, precipitation regions appeared as sharp peaks, and the inverted gray value showed spatial periodicity. Furthermore, the interval between two successive peaks increased with the distance from the gel surface. Therefore, the regular-type periodic precipitation pattern (LP) was formed. The baseline oscillated in space around *x* = 30–45 mm at φ_DMSO_ = 0.2, which indicated the presence of two types of regions having different amounts of precipitate, as seen in Figure 1a. Furthermore, broad peaks (e.g., at *x* = 18–25 mm) appeared periodically throughout the pattern at φ_DMSO_ = 0.3, and a high-frequency pattern was superimposed on the low-frequency pattern (Figure 2a(i)). One low-frequency zone contained ∼10 precipitation bands from the high-frequency pattern. With further increase of φ_DMSO_, the amplitude of the oscillation of the high-frequency pattern decreased (φ_DMSO_ = 0.4, Figure 2a(ii)), and the high-frequency pattern disappeared, only the low-frequency revert pattern dominated at φ_DMSO_= 0.5. Also, we analyzed the variation of the width of *n*th band (*w*_*n*_) in *x*_*n*_ for the revert-type bands (low-frequency patterns) obtained at φ_DMSO_ = 0.3–0.5 (Figure S10 in the SI). Previous experiments and simulations showed that *w*_*n*_ usually increases linearly with increasing *x*_*n*_ (*w*_*n*_ ∼ *x*_*n*_: so-called width law) for the regular-type LPs, which means that the density of material in each band is constant, and they satisfy the mass conservation.^48,49^ In contrast, the obtained revert patterns in this study showed a linear decrease in *w*_*n*_ with increasing *x*_*n*_, which indicates that the revert-type LPs should satisfy the inverted form of the classical width law for the regular-type LPs.

Profiles of the patterns obtained using other solvents also showed a good agreement with the trend obtained in DMSO (Figure S11 in the SI). In particular, EG had a very similar pattern of transition behavior. We can conclude that lowering the polarity of the gel medium leads to a global pattern transition from the regular to the revert one with a hierarchical structure, namely the coexistence of high and low-frequency precipitation regions. Figure 2c shows the relationship between the spacing coefficient (eq 1) and φ_DMSO_, where *p* is calculated from the primary regular-type at φ_DMSO_ = 0–0.1 and the high-frequency pattern above φ_DMSO_ = 0.2. The spacing coefficient decreases exponentially with the DMSO content. Furthermore, this change showed a good agreement with a fitting curve based on the power function. Other solvents also showed a similar trend (Figure S12 in the SI), the decreasing polarity reduces the inter-band spacing of regular LPs and facilitates the formation of revert hierarchical precipitation structures.

**Figure 3:**
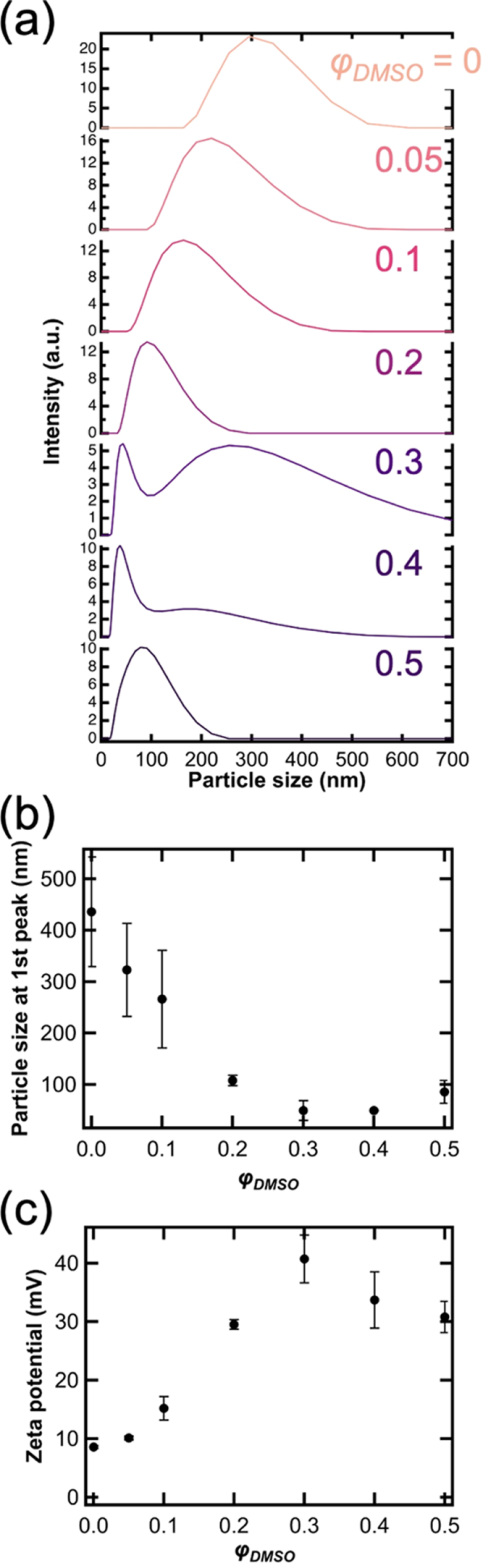
(a) Size distribution of CuCrO_4_ colloidal particles in aqueous solution with different φ_DMSO_, measured by dynamic light scattering (DLS). (b) Relationship between the mean particle size at the 1st peak (with a smaller size in bimodal distribution) and φ_DMSO_. (c) Relationship between the ζ potential of CuCrO_4_ particles and φ_DMSO_. Error bars were obtained from 3 replicates, which were calculated using *p*-values < 0.05.

To obtain more information about the possible mechanism of the pattern transition and the formation of low-frequency bands, we measured the particle size and ζ potential of CuCrO_4_ particles formed in water/DMSO mixtures with different φ_DMSO_. Figure 3a shows the size distribution measured by dynamic light scattering (DLS). When φ_DMSO_ = 0, a rather broad peak centered at 300 nm is obtained, which narrows and decreases until φ_DMSO_ = 0.2. At φ_DMSO_ = 0.3 and 0.4, a bimodal distribution appeared, the value of the smaller size peak was less than 100 nm, the second one is greater than 200 nm. The size at the 1st peak (corresponding to smaller particles) decreased monotonically with φ_DMSO_ and slightly increased at φ_DMSO_ = 0.5 (Figure 3b). At the same time, the ζ potential shows a pronounced increase with increasing DMSO content (Figure 3c). Particles had a positive ζ potential (∼10 mV) in water (φ_DMSO_ = 0), indicating excess Cu^2+^ ion adsorption at the surface of the CuCrO_4_ particles. The measured ζ potential peaked around 40 mV at φ_DMSO_ = 0.3, while a further increase of the DMSO content decreases its value. The reason why the ζ potential increased up to φ_DMSO_ = 0.3 is that the decreasing dielectric constant of the embedding medium leads to improvement of electric repulsion because ε_r_ is in the denominator in the relationship of electrostatic interaction.^50^ Furthermore, it has to be noted that the ζ potential increase is accompanied by decreasing DLS size (Figure 3a,b), which indicates a more stable nanoparticle dispersion as a result of increased electric double-layer repulsion. The apparent decrease of the ζ potential above φ_DMSO_ = 0.3 can be attributed to the loss of particle stability, also reflected in the appearance of a second peak on the DLS graph. On the other hand, as with decreasing solvent polarity the attractive dispersion interaction increases rapidly,^50^ after a given DMSO content, an increase in attraction will dominate, resulting in the partial loss of colloid stability, hence a larger DLS size. Similar measurements were carried out for DMF, EG, and TBA as well (Figure S13 in the SI), leading qualitatively to the same results. It has to be pointed out, that the solvent composition where particle stability is lost, correlates with the dielectric constant of each added solvent; the lower its value, the smaller volume fraction, which is sufficient to induce the formation of a population with a larger size. This bimodal distribution has important implications for the formation of complex patterns in the gels, as in this case, two different precipitation processes can take place with different kinetics.^28,29,51^ Also, this bimodal distribution appeared only in the presence of agarose (Figure S14a,b in the SI). Furthermore, the existence of two different precipitation processes was strongly supported by the kinetics of the particle size measurements in the water/DMSO mixture with a low concentration of agarose (Figure S14c in the SI), where the rate of increase in the hydrodynamic size of the peak for bigger particles in the bimodal distribution was greater than that for the peak for smaller particles due to the electrostatic stabilization. Therefore, this distribution was likely to result from a relatively fast precipitation process (heterogeneous precipitation) facilitated by the presence of agarose for unstable particles due to the decrease in polarity and a relatively slow process, in which the remaining electrostatically stabilized particles themselves form precipitates (homogeneous precipitation). Based on this, a possible mechanism to explain why the hierarchical structure was formed with decreasing polarity is proposed as follows. Since a major component of the system at φ_DMSO_ = 0 is unstable colloidal particles with low ζ potential, the heterogeneous process dominates the precipitation and the high-frequency pattern formation. However, the proportion of electrostatically stable particles increases with increasing φ_DMSO_, the contribution of the homogeneous process increases, and the system creates bimodal distribution (i.e., the competition between the heterogeneous and homogeneous process), which induces the formation of low-frequency and hierarchical patterns. Furthermore, because the peak with bigger particles finally unified with the peak of smaller particles, and the bimodal distribution disappeared at φ_DMSO_ = 0.5, the homogeneous process becomes the dominant process in the pattern formation mechanism instead of the heterogeneous process, which induces localizing and reducing the abundance of high-frequency pattern as shown in Figures 1b, 2a,b, and S2 in the SI.

We also performed numerical simulations to verify the validity of the above possible mechanism. As we mentioned above, we considered the homogeneous and heterogeneous precipitation processes, where nucleation and aggregation of a small-size species take place in the solvent phase (homogeneous precipitation), with the eventual deposition of the aggregates on agarose, which itself acts as a source of heterogeneous precipitation as well. The latter allows for the formation of large species directly on the biopolymer matrix. We assumed that a competition of these two processes forms both the high and low-frequency patterns in the system. The following mechanism was used in the simulations:

Homogeneous precipitation process:
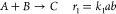
3
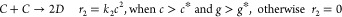
4
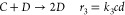
5
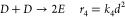
6

Heterogeneous precipitation process:
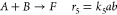
7
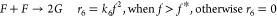
8
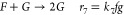
9
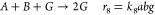
10

The reaction between Cu^2+^ (*A*) and CrO_4_^2–^ (*B*) produces seeds (*C*) (eq 3), and *C* turns into bigger nanoparticles (*D*) by growth and aggregation (eq 4) and autocatalytic aggregation (eq 5), *D* finally transforms into an immobile precipitate (*E*) (eq 6). In a competitive process, the seeds attached to the agarose network (*F*) are formed through heterogeneous nucleation (eq 7), and precipitate (*G*) is directly formed from *F* by aggregation and precipitation because the diffusion of particles is limited on the agarose matrix (eq 8). Equations 9 and 10 represent autocatalytic aggregation and precipitation. *r*_1_–*r*_8_ are the reaction rates for each process, and *k*_1_–*k*_8_ are the corresponding rate constants. *a*, *b*, *c*, *d*, *e*, *f*, and *g* are the concentrations of *A*, *B*, *C*, *D*, *E*, *F*, and *G*, respectively. c*, f*, and g* are the threshold concentrations. These processes can be expressed by the following set of partial differential equations (reaction-diffusion equations)
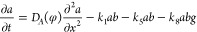
11
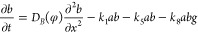
12
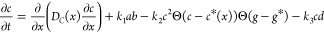
13
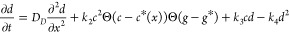
14
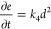
15
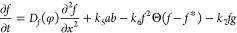
16
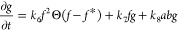
17where *D*_*A*_, *D*_*B*_, *D*_*c*_, *D*_*D*_, and *D*_*F*_ are the diffusion coefficients of *A*, *B*, *C*, *D*, *E*, *F*, and *G*, respectively. In the model, *D*_*A*_, *D*_*C*_, *D*_*F*_, and c* are expressed as functions of the volume fraction (φ) and distance (*x*) based on the experimental results (for details see the SI) to relate the polarity effect to the reaction-diffusion kinetics in the Liesegang system with CuCrO_4_ precipitates. Briefly, *D*_*A*_(φ) and *D*_*F*_(φ) were expressed as decreasing functions of φ. In fact, the diffusion rate of Cu^2+^ was strongly decelerated by increasing φ (Figure S15 in the SI), while the ζ potential increased with φ (Figure 3c), revealing a stronger interaction of the particles with the agarose matrix. On the other hand, the polarity dependence of *D*_*F*_ was not applied to *D*_*D*_ since *D* was defined as one of the chemical species for the homogeneous process, and there is no electrostatic interaction between *D* and agarose. Due to the diffusion of the outer electrolyte (CuCl_2_) into the gel, the concentration of the Cu^2+^ ions decreases as we go away from the interface. The lower copper ion concentration decreases the surface charge, which results in less stable particles (c* decreases). Also, some previous studies showed that the particle size increased in the distance measured from the gel–liquid interface in Liesegang systems,^52^ we expected that a decrease in c* also led to increasing the particle size. In summary, this effect might have two consequences for the CuCrO_4_ particles without binding to the agarose: (i) the diffusion coefficient of *C* decreases due to the increase in particle size, and (ii) the aggregation threshold decreases in *x*. Therefore, we expressed both *D_C_* and c* as decreasing functions of *x* as discussed in some previous studies.^21−23,25,26^ Furthermore, these studies showed that the decreasing function of threshold was mainly contributed by revert LP. Therefore, we predicted that the low-frequency revert LP emerged when the homogeneous process is pronounced by the stabilization of particles due to the increase in φ. In contrast to the expression of *D*_*C*_, we applied that *D*_*D*_ was independent of the position (*x*). According to the previous study which investigated the diffusivity of nanoparticles in a gel medium, the diffusion coefficient of particles decreased inversely with the hydrodynamic radius.^53^ This result indicates that differences in diffusion coefficients due to size differences between relatively large particles are not significant. Since *D* corresponds to bigger particles than *C*, the size effect on the diffusivity of *D* should be less than *C*. Therefore, we used the expression without the dependency of position for *D*_*D*_. The details of the numerical model and the simulations (including information for all parameters) can be found in the SI.

**Figure 4:**
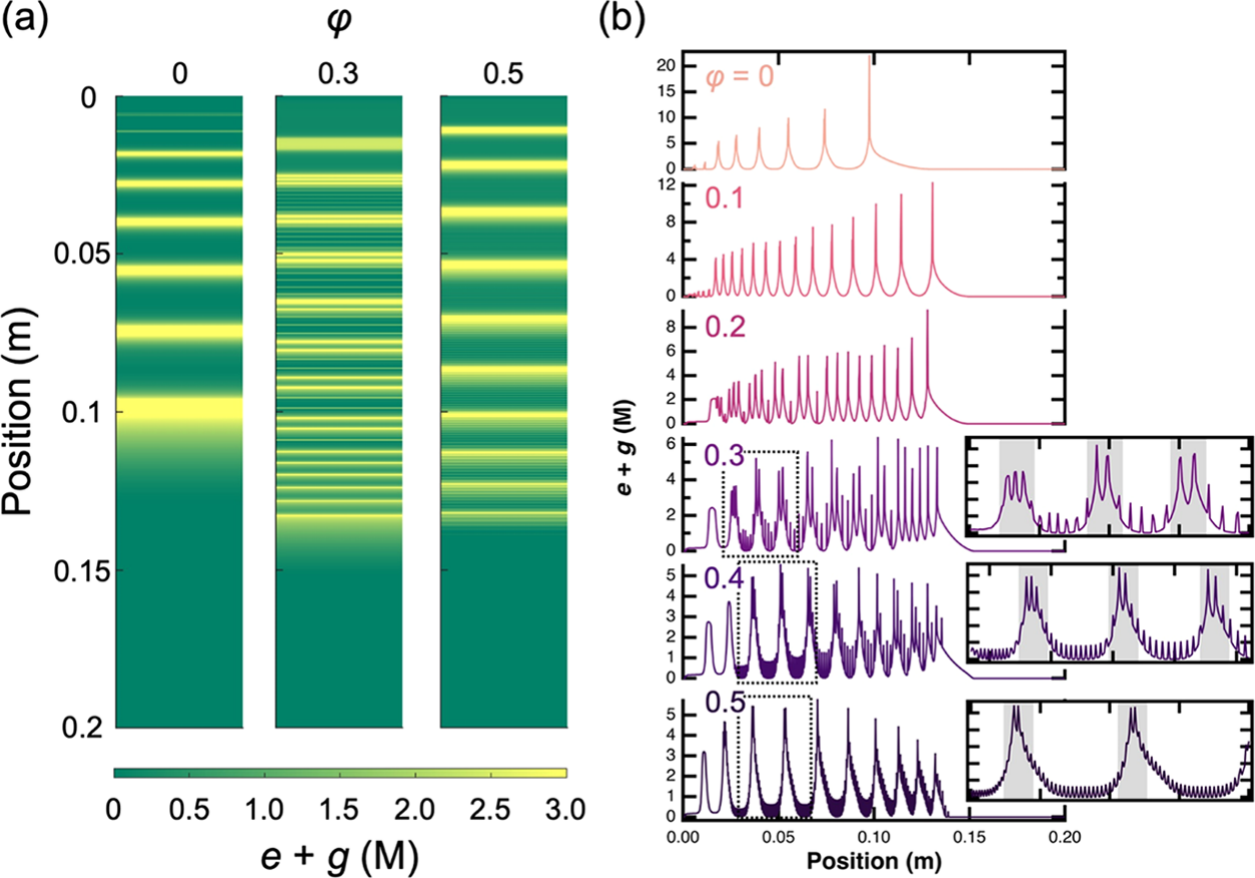
(a) Effect of φ on the pattern structure in simulations (φ_DMSO_ in experiments), where φ = 0 and 0.5 correspond to conditions of pure water and water/DMSO mixture of 1:1. Color changes from green to yellow show the sum of concentrations (*e* + *g*) of species *E* and *G*. (b) Concentration profiles of *E* + *G* in simulations at various φ. Regions surrounded by the dotted line square at φ = 0.3–0.5 are inserted into the right panel of each condition as enlarged graphs. Gray regions in enlarged graphs correspond to the formation of the low-frequency revert patterns.

The simulation results provided a qualitative agreement with the results of global transition and formation of high and low-frequency patterns observed in the experiments (Figures 4 and S16 in the SI). When φ = 0, the regular LP was formed (Figure 4a left panel), the same as in the experiment at φ_DMSO_ = 0. As φ was increased, the frequency of the primary patterns increased and the low-frequency pattern appeared generating a superimposed high-frequency pattern on the low-frequency one (Figure 4a middle panel). In the case of φ = 0.5, the high-frequency pattern disappeared completely in the system leaving only low-frequency revert patterns (Figure 4a right panel). These trends become more vivid by obtaining concentration profiles of *E* and *G* (Figure 4b). This result shows a good agreement with the experimental results (Figures 2b and S2 in the SI). Also, the contribution of each process is shown in Figure S17 in the SI, where the homogeneous process formed low-frequency revert LPs with increasing φ, while the heterogeneous process promoted high-frequency regular LP formation. Therefore, based on the simulation results, we can propose that the pattern transitions and the formation of the superimposed high and low-frequency patterns are driven by the polarity of the solvent and governed by two precipitation pathways with different reaction rates.

## 4 Conclusions

In summary, in experiments on the precipitation of CuCrO_4_ in different mixtures of water and various organic solvents (DMSO, DMF, EG, TBA, and GC), we have demonstrated that the polarity of the medium in a hydrogel matrix affects the morphology of LP. We interpreted the observed results with the support of numerical simulation based on the homogeneous and heterogeneous precipitation processes. Typically, depending on the DMSO content (i.e., overall polarity of the solvent mixture), (i) we could control the frequency of regular LPs (Figure 2c), and (ii) the global periodicity transited from regular to revert patterns, we finally obtained the hierarchical pattern coexisting with the revert low-frequency pattern with superimposed high-frequency regular pattern (Figures 1b and 2b). Similar trends were also observed in some other organic solvents as well. The results of size and ζ potential measurements showed the possibility that the competition of two different precipitation processes was induced depending on the polarity of the medium. Based on this result, we constructed one possible model, and simulation using this model showed good qualitative agreement with experimental observations. According to this behavior, LP could be employed for practical purposes to track the polarity changes in unknown solvents, similar to the use of LPs for tracking environmental changes.^15,16,30^ Moreover, tuning the solvent polarity can be a promising strategy to control, tailor, and design both regular LPs and new hierarchical structures. In addition, hierarchical layered structures are of great importance for the design of self-organized functional materials from nano- to macroscale.^3−5^ Some recent studies showed that LPs can be formed using some functional materials (e.g., metal nanoparticles and metal–organic frameworks), and another study indicated how to form microscale periodicity.^17,20,54,55^ By combining this knowledge with our strategy of polarity-based LP control, LP could be a potential candidate for the generation of self-organized hierarchical layered structures with various functions and structures.

## Supporting Information Available

Procedures for the synthesis of GC, solubility product (*K*_sp_) measurements, and line profile analysis, detail of numerical simulation and model; results of optical microscopy observations, time-course, pattern formation with organic solvents (DMF, EG, TBA, GL, and GC), line profile, spacing coefficient, DLS, and ζ potential measurements, *K*_sp_ measurements, diffusion of Cu^2+^, and simulations (PDF)

## Abbreviations

LP: Liesegang pattern
EG: ethylene glycol
GL: glycerol
DMF: *N*,*N*-dimethylformamide
DMSO: dimethyl sulfoxide
TBA: *tert*-butyl alcohol
DLS: dynamic light scattering
ε_r_: relative dielectric constants
