# Supplementary material for: Effect of the Polarity of Solvents on Periodic Precipitation: Formation of Hierarchical Revert Liesegang Patterns
Source: J Phys Chem B. 2022 Oct 11;126(41):8322–30. doi: 10.1021/acs.jpcb.2c05810 (PMC9589725; doi:10.1021/acs.jpcb.2c05810)
Supplement: Supplementary file 1 — jp2c05810_si_001.pdf [file jp2c05810_si_001.pdf]

Supporting Information

Effect of the Polarity of Solvents on a Periodic Precipitation: Formation of Hierarchical Revert Liesegang Patterns

Gábor Holló,[a] Dániel Zámbó,[b] András Deák,[b] Federico Rossi,[c] Raffaele Cucciniello,[d] Pierandrea Lo Nostro,[e] Hideki Nabika,[f] Bilge Baytekin,[g] István Lagzi,[a,h]* and Masaki Itatani [h]*

[a] ELKH-BME Condensed Matter Research Group, Budapest University of Technology and Economics, Műegyetem rkp. 3, Budapest 1111, Hungary

[b] Institute of Technical Physics and Materials Science, Centre for Energy Research, Konkoly-Thege M. út 29-33, H-1121 Budapest, Hungary

[c] Department of Earth, Environmental and Physical Sciences - DEEP Sciences, University of Siena, Pian dei Mantellini 44, 53100 - Siena, Italy

[d] Department of Chemistry and Biology "Adolfo Zambelli", University of Salerno, Via Giovanni Paolo II, 132, 84084 Fisciano, Salerno, Italy

[e] Department of Chemistry ''Ugo Schiff", University of Florence, Via della Lastruccia 3, 50019 Sesto Fiorentino, FI, Italy

[f] Faculty of Science, Yamagata University, 1-4-12, Kojirakawa, Yamagata 990-8560, Japan

[g] Department of Chemistry and UNAM, Bilkent University, 06800, Ankara, Turkey

[h] Department of Physics, Institute of Physics, Budapest University of Technology and Economics, Budafoki út 8, Budapest 1111, Hungary

Corresponding author*

*(I. Lagzi) lagzi.istvan.laszlo@ttk.bme.hu, *(M. Itatani) masakiitatani.chem@gmail.com

Table of Contents

1 Experiments

1.1 Synthesis of glycerol carbonate (GC)

1.2 Determination of solubility product (K_{sp}) by UV-vis measurements 3

1.3 Line profile analysis 3

2 Numerical model and simulations 4

3 Results and Discussion

3.1 Optical microscopy observations 9

3.2 Time course of pattern formation 10

3.3 Pattern formation with other solvents (DMF, EG, TBA, GL, and GC) 13

3.4 Width law for the revert-type LPs (low-frequency patterns) 17

3.5 Line profile analysis 18

3.6 Relationship between the spacing coefficient (p) and φ_{OS} 19

3.7 DLS and zeta potential measurements 20

3.8 Effect of φ_{OS} on diffusion of Cu^{2+} 21

3.9 Simulations 24

3.10 K_{sp} measurements 26

4 References 27

1 Experiments:

1.1 Synthesis of glycerol carbonate (GC)

GC was synthesized in mild reaction conditions through glycerol transcarbonation in the presence of dimethyl carbonate (DMC). In detail, 450 g (5.0 mol) of DMC, 150.3 g of GL (1.67 mol) and 0.53 g of Na_2CO_3 were introduced into a 2 L three-neck flask equipped with a mechanical stirring, a system condenser and a temperature sensor. The mixture was stirred and refluxed (T = 75°C) for 2 h, then the catalyst was filtered off and the excess DMC and the methanol formed as a by-product were distilled under reduced pressure. GC was obtained as a colorless viscous liquid with a purity of 96%, (determined by NMR analyses). The NMR spectra were collected on Bruker Avance-400 (100 ^{13}C) spectrometer using deuterated DMSO as solvent. 13C-NMR (100 MHz, DMSO): 155.2 (CO), 77.1 (CH), 65.92 (CH_2), 60.65 (CH_2)

1.2 Determination of solubility product (K_{sp}) by UV-vis measurements

Firstly, the water/organic solvents mixture with K_2CrO_4 was prepared in a microtube. Subsequently, the $CuCl_2$ solution was added to this mixture and mixed by repeating pipetting up and down 10 times. The concentrations of K_2CrO_4 and $CuCl_2$ were fixed at 1.0 × 10^{-2} M, only φ_{OS} was changed by adjusting the mixing volume ratio between water and organic solvents to investigate the effect of φ_{OS} on K_{sp} values of $CuCrO_4$. After addition of the $CuCl_2$ solution, this mixture was allowed to stand for 10 min to complete the reaction between Cu^{2+} and CrO_4^{2-}. Subsequently, this solution was centrifugated to precipitate all colloidal particles of $CuCrO_4$ from the solution, and then obtained supernatant was subjected to UV-vis measurements. After the measurement, the concentration of CrO_4^{2-} was determined from a pre-prepared calibration curve for absorbance at λ = 370 nm. The reaction between Cu^{2+} and CrO_4^{2-} is denoted as the following chemical equation.

$$Cu^{2+} + CrO_4^{2-} \rightarrow CuCrO_4 \qquad (1)$$

Since the stoichiometric ratio of Cu^{2+} to CrO_4^{2-} is 1:1, and the solubility product can be calculated as:

$$[Cu^{2+}][CrO_4^{2-}] = K_{sp}. \qquad (2)$$

Therefore, we could calculate K_{sp} by substituting the above concentration values to this relationship.

1.3 Line profile analysis

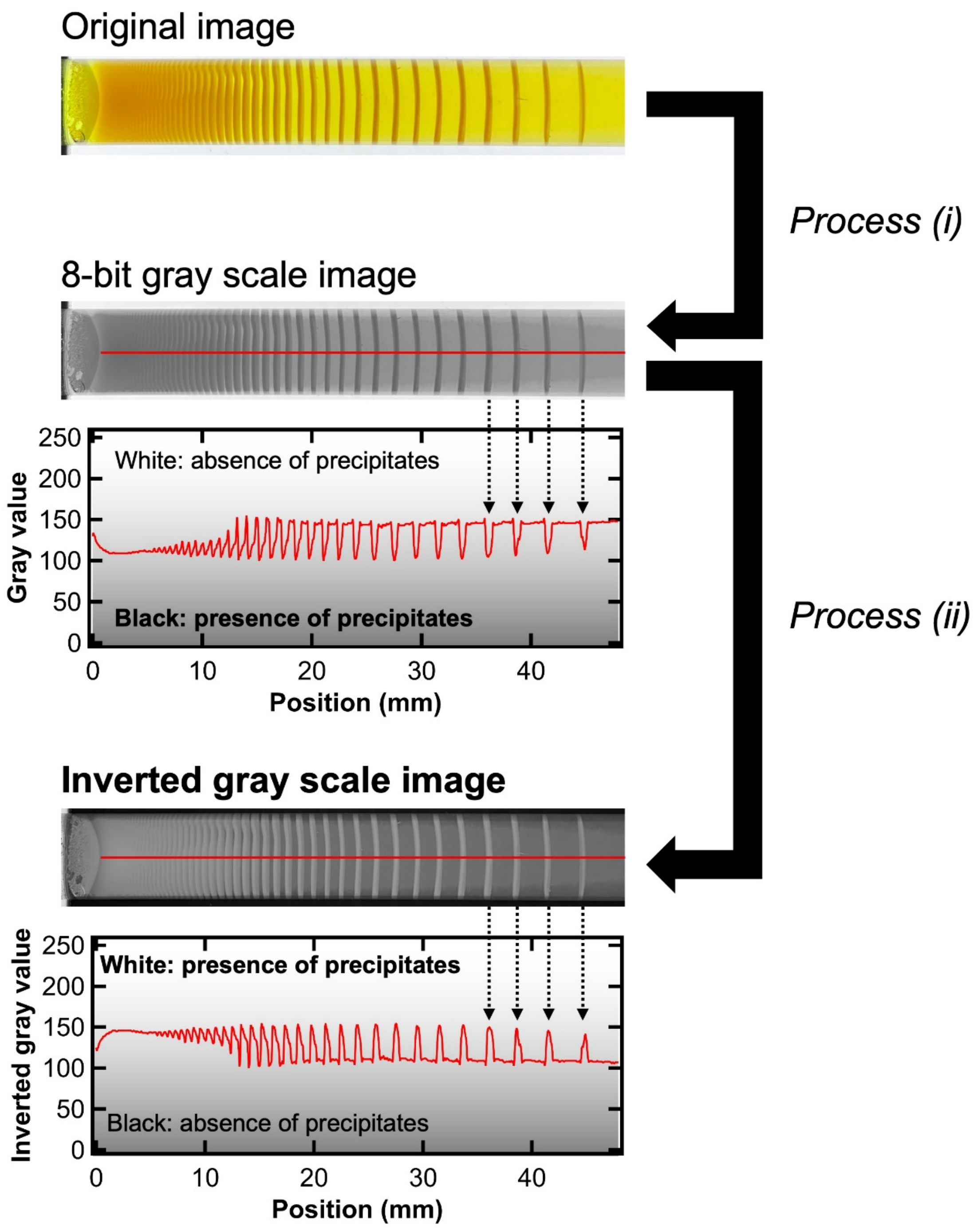

Figure S1. Scheme of line profile analysis in this study. *Process* (i): Original image taken by a digital camera was converted to an 8-bit grayscale image by using the Image J software. *Process* (ii): Inverted gray scale image was obtained by inverting the gray value in the 8-bit grayscale image. These processes allow the position of precipitation to be recognized more intuitively than in simple profile results (*process* (i)), as the precipitation position is represented as a "peak" in the line profile analysis results with inverted gray values.

2 Numerical model and simulations

We constructed a reaction–diffusion (RD) model by combining the homogeneous and heterogeneous precipitation processes to describe the experimentally observed transition of LP depending on the solvent composition (φ). In the case of heterogeneous precipitation, the processes proceed with the species connected to the agarose network, while the homogeneous precipitation takes place in the solvent phase. We assumed that a competition of these two processes forms both the primary and secondary patterns in the system. The following mechanism was used in the simulations:

Homogeneous precipitation process:

A + B → C	$r_1 = k_1 ab$,	(3)
C + C → 2D	$r_2 = k_2 c^2$, when $c > c^*$ and $g > g^*$, otherwise $r_2 = 0$,	(4)
C + D → 2D	$r_3 = k_3 cd$,	(5)
D + D → 2E	$r_4 = k_4 d^2$,	(6)

Heterogeneous precipitation process:

A + B → F	$r_5 = k_5 ab$,	(7)
F + F → 2G	$r_6 = k_6 f^2$, when $f > f^*$, otherwise $r_6 = 0$	(8)
F + G → 2G	$r_7 = k_7 fg$,	(9)
A + B + G → 2G	$r_8 = k_8 abg$,	(10)

The reaction of the Cu^{2+} (A) and CrO_4^{2-} (B) ions in the solvent phase produces seeds (C), which in the homogeneous process, grow into bigger nanoparticles (D) and they aggregate to an immobile precipitate or aggregate (E). In the heterogeneous precipitation, the formed seeds (F) and the precipitate (G) are attached to the agarose network. Equations (3) and (7) represent the nucleation. In the homogeneous process, C turns into D by the aggregation (eq. (4)) and autocatalytic aggregation (eq. (5)), then D is precipitated by further aggregation and precipitation (eq. (6)). Also, for the heterogeneous process, F is directly translated into precipitates G by aggregation and precipitation because the diffusion of large particles is inhibited on the agarose matrix (eq. (8)). Equations (9) and (10) represent autocatalytic aggregation and heterogeneous precipitation. Also, r_1–r_8 represent chemical rate laws for each process, and k_1–k_8 are reaction rate constants corresponding to each process. a, b, c, d, e, f, and g are the concentrations of A, B, C, D, E, F, and G, respectively. c^*, f^*, and g^* are the threshold concentrations that we will explain in detail later. These processes can be expressed by the following set of partial differential equations (RD equations), which were used for performing simulations in one dimension (along the gel column):

$$\frac{\partial a}{\partial t} = D_A(\varphi)\frac{\partial^2 a}{\partial x^2} - k_1 ab - k_5 ab - k_8 abg, \quad (11)$$

$$\frac{\partial b}{\partial t} = D_B(\varphi)\frac{\partial^2 b}{\partial x^2} - k_1 ab - k_5 ab - k_8 abg, \quad (12)$$

$$\frac{\partial c}{\partial t} = \frac{\partial}{\partial x}\left(D_C(x)\frac{\partial c}{\partial x}\right) + k_1 ab - k_2 c^2 \Theta\big(c - c^*(x)\big)\Theta(g - g^*) - k_3 cd, \quad (13)$$

$$\frac{\partial d}{\partial t} = D_D \frac{\partial^2 d}{\partial x^2} + k_2 c^2 \Theta\big(c - c^*(x)\big)\Theta(g - g^*) + k_3 cd - k_4 d^2, \tag{14}$$

$$\frac{\partial e}{\partial t} = k_4 d^2, \tag{15}$$

$$\frac{\partial f}{\partial t} = D_f(\varphi) \frac{\partial^2 f}{\partial x^2} + k_5 ab - k_6 f^2 \Theta(f - f^*) - k_7 fg, \tag{16}$$

$$\frac{\partial g}{\partial t} = k_6 f^2 \Theta(f - f^*) + k_7 fg + k_8 abg, \tag{17}$$

where D_A, D_B, D_c, D_D, and D_F are the diffusion coefficients of A, B, C, D, E, F, and G, respectively. E and G are precipitate species, therefore, they are immobile ($D_E = D_G = 0$). Since K_{sp} was almost constant in the case of DMSO (Figure S16) and this value is practically much smaller than a threshold concentration of aggregation, K_{sp} is therefore not included in our model. To reflect the experimental observation that the diffusion coefficient of Cu^{2+} decreases with the ratio of DMSO in the solvent (Figure S1), we introduced a linear function for D_A and D_B:

$$D_A(\varphi) = D_B(\varphi) = 10^{-9} - 1.6 \times 10^{-9}\varphi \ \ (\mathrm{m^2\,s^{-1}}). \tag{18}$$

A previous study showed that the interaction energy of water–DMSO is higher than water–water.[1] Namely, increasing φ leads to a decrease in the number of hydration water molecules to ions. The unhydrated ions interact stronger with ionic residues of the agarose chain by electrostatic and non-electrostatic (e.g. dispersion) interactions, which contribute to binding the molecules in the gel matrix where the agarose is present, reducing their mobility. Therefore, increasing φ causes stronger interaction between ions and agarose molecules due to intercepting hydration molecules from ions, and decreasing diffusivity. Also, from the zeta potential measurements (Figure 3c), it was suggested that Cu^{2+} adsorbs at the surface of the particles since the $CuCrO_4$ particle had a positive charge. In our Liesegang system, a Cu^{2+} concentration gradient was also formed during the precipitation process, therefore, we hypothesized that the amount of adsorption decreases as we go farther from the interface between Cu^{2+} solution and gel, and we have a surface charge gradient of particles in the system. The particle should be more unstable further away from the solution-gel interface according to the surface charge gradient, growth of them is promoted. Thus, the size of particle increases as we go further away from the interface.[2] This effect causes a gradient in the particle diffusivity. In other words, a gradient in the diffusion coefficient of seeds should be formed, where the coefficient decreases with the distance (x) from the interface. This hypothesis is represented in the following function of D_C:

$$D_C(x) = D_A(\varphi) \times \left(1 - \frac{0.9}{L} x\right) \ \ (\mathrm{m^2\,s^{-1}}). \tag{19}$$

where L is the domain length of the system. Different from C, F is strongly adsorbed on the surface of the agarose network. Therefore, it should be less affected by the effect of Cu^{2+} adsorption due to the Cu^{2+} gradient as described earlier, and its diffusivity should depend only on the degree of its interaction with the agarose molecule, namely, it should be described as a function of φ, not as a function of x. Since zeta potential increases with the increase of

φ_{DMSO}, the interaction of particles (F) with agarose is enhanced with φ. Therefore, the diffusion coefficient of F decreases with φ:

$$D_F(\varphi) = D_A(\varphi) \times 10^{-(1+4\varphi)} \ (\mathrm{m^2\,s^{-1}}). \quad (20)$$

It is noted that D_F is at most one-tenth of D_A when $\varphi = 0$ (pure water), whereas D_C is set to be equivalent to D_A at $x = 0$ (eq. (19)). This is because we consider the diffusivity of F is reduced originally due to the adsorption on the agarose matrix. The diffusion coefficient of D was set as a fixed value at $D_D = D_A(\varphi)/10000\ \mathrm{m^2\,s^{-1}}$. To consider the kinetics of all processes (eqs. (3)–(10)), the rate constants (k) for each process were adjusted. k_1–k_8 in RD equations correspond to eq. (3) to (10), in that order. In particular, the sequence of processes from nucleation to precipitation is generally faster for heterogeneous processes, therefore, k_6–k_8 were set higher than k_2–k_4. The processes of A + B $\rightarrow$ C (eq. (3)) and A + B $\rightarrow$ F (eq. (7)) involve reaction and nucleation processes. Essentially, a relationship of k_1 and k_2 should be $k_1 < k_2$, however, we have to consider the numerical ratio of these processes. Due to the low space ratio occupied by agarose, $k_1 > k_2$ was adopted for our model because eq. (3) driving the homogeneous process is expected to be a higher proportion of the total reaction. All values of k_1–k_8 are denoted following; $k_1 = 10^{-4}\ \mathrm{M^{-1}\,s^{-1}}$, $k_2 = 10^{-5}\ \mathrm{M^{-1}\,s^{-1}}$, $k_3 = 10^{-4}\ \mathrm{M^{-1}\,s^{-1}}$, $k_4 = 10^{-4}\ \mathrm{M^{-1}\,s^{-1}}$, $k_5 = 5 \times 10^{-6}\ \mathrm{M^{-1}\,s^{-1}}$, $k_6 = 10^{-3}\ \mathrm{M^{-1}\,s^{-1}}$, $k_7 = 10^{-2}\ \mathrm{M^{-1}\,s^{-1}}$, and $k_8 = 10^{-2}\ \mathrm{M^{-2}\,s^{-1}}$, respectively. In our experiments, we considered that the aggregation of nanoparticles (eqs. (4) and (8)) took place only when their concentrations (C and F) exceed given threshold concentrations, this approach is known as the sol-coagulation model in the Liesegang system.[3] To reflect it in the simulations, we used the Heaviside step function (Θ) in RD equations. c^* and f^* are the threshold concentrations for the formation of D and G through the aggregation of nanoparticles in homogeneous and heterogeneous processes. To guarantee the kinetic advantage of the heterogeneous process, we also introduced a new threshold g^* for the aggregation process of the homogeneous process (eq. (4)). The existence of $\Theta(g - g^*)$ in eq. (4) represents that C + C $\rightarrow$ 2D (the homogeneous precipitation process) can occur only if G (precipitates from the heterogeneous precipitation process) already exists. The threshold values of f^* and g^* are 0.05 and 0 M, respectively. Since seeds for the homogeneous process (C) have the spatial gradient of surface potential as we mentioned earlier, threshold c^* is treated differently from other thresholds. In our simulations, we hypothesized that the surface charge of C was decreased by moving farther from the liquid–gel interface (*i.e.*, C can be aggregated easier farther from the solution–gel interface). To introduce this effect, we used a space-dependent function of c^*:

$$c^*(x) = 0.7 - \frac{0.6}{L}x \ (\mathrm{M}). \quad (21)$$

The initial and boundary conditions were set to represent the experimental conditions, namely, $a(t = 0, x) = c(t = 0, x) = d(t = 0, x) = e(t = 0, x) = f(t = 0, x) = g(t = 0, x) = 0$ M, $b(t = 0, x) = 1$ M (fixed in all simulations). We used no-flux boundary condition at the end of the domain ($x = L$) and the Dirichlet boundary condition at the liquid–gel interface ($x = 0$) for all species: $a(t, x = 0) = 10$ M (fixed in all simulations), $b(t, x = 0) = c(t, x = 0) = d(t, x = 0) = e(t, x = 0) = f(t, x = 0) = g(t, x = 0) = 0$. The length of the simulation domain, grid spacing, simulation time, and the time step were $L = 0.2$ m, $\Delta x = 2 \times 10^{-4}$ m, $T = 2 \times 10^7$ s, and $\Delta t = 2$ s, respectively. All simulations were

carried out by using MATLAB software, and the differential equations were solved with the Forward Time Centered Space (FTCS) method.

3 Results and Discussion

3.1 Optical microscopy observations

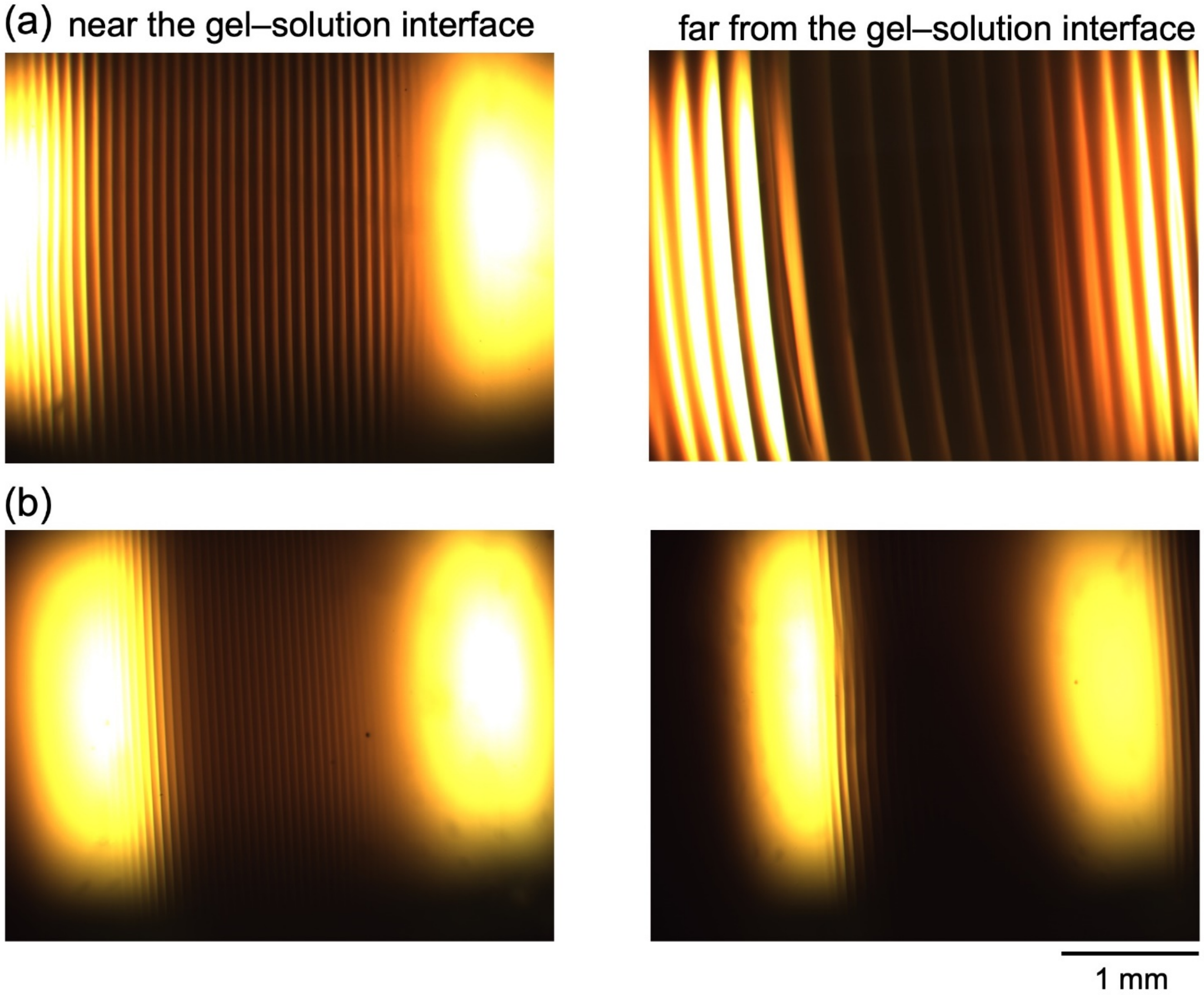

Figure S2. Microscope images at (a) φ_{DMSO} = 0.3 and (b) 0.5. Images taken near the solution-gel interface are shown at the left in both (a) and (b), whereas the position near the bottom of glass tube are shown at the right. The darker and brighter regions correspond to the primary band position and the inter-band space.

3.2 Time course of pattern formation

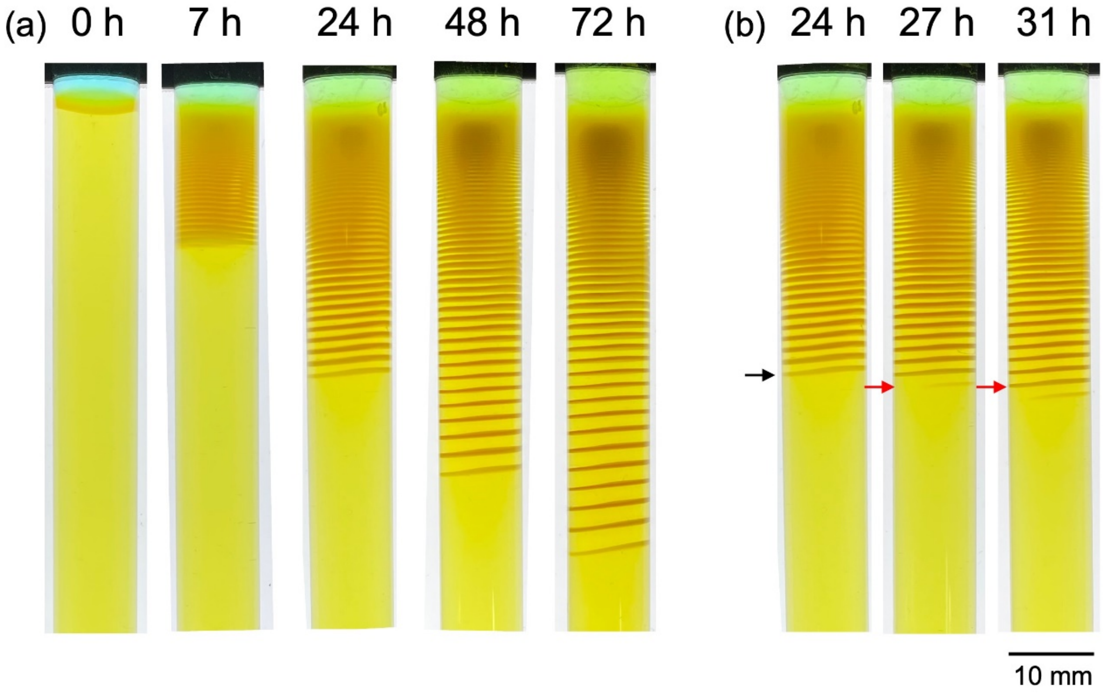

Figure S3. (a) Time course of pattern formation at φ_{DMSO} = 0 from 0 h to 72 h. (b) More detailed evolution to form one set of a high-density and low-density of precipitates regions.

After the earlier precipitates band formation (black arrow), a part of the next band (red arrow) appears in the gel after a certain distance corresponding to the low-density of precipitates region. Then, the process is completed by growing it. The formation of band and inter-band space proceed at the same time.

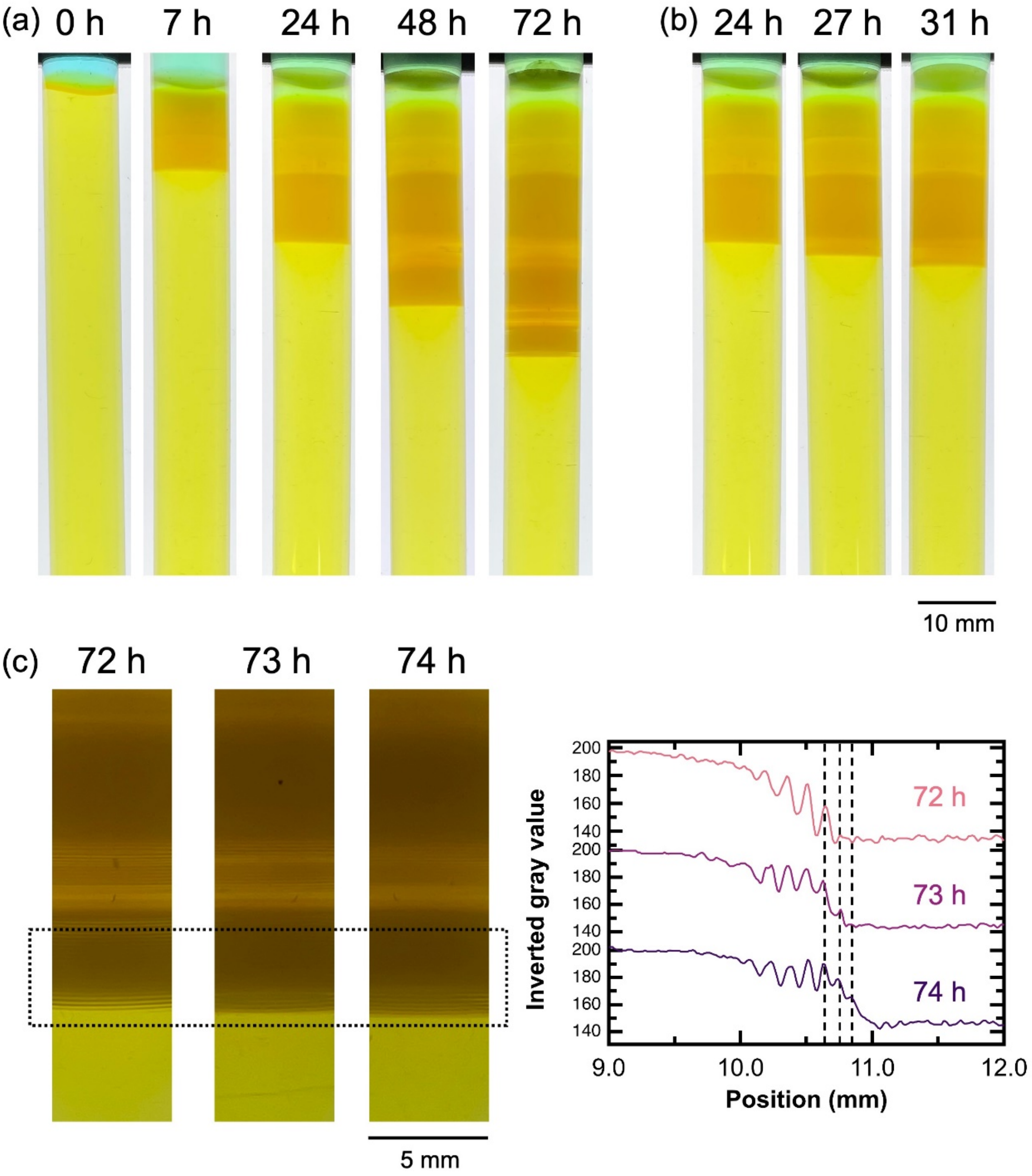

Figure S4. (a) Time course of pattern formation at φ_{DMSO} = 0.3 from 0 h to 72 h. (b) More detailed evolution to form a space between primary inverted precipitate bands. (c) Time course of formation of a high-frequency pattern near a precipitation front of low-frequency pattern (left), and line profile analysis at the region surrounded by the dotted line square (right), where dashed lines in the profile represent positions of bands.

After the earlier band formation, a low-density precipitates region that is corresponding to the inter-band space is gradually formed. A set of a high-density and low-density of precipitates regions for the high-frequency pattern is formed with a similar process to the case of $\varphi_{DMSO} = 0$.

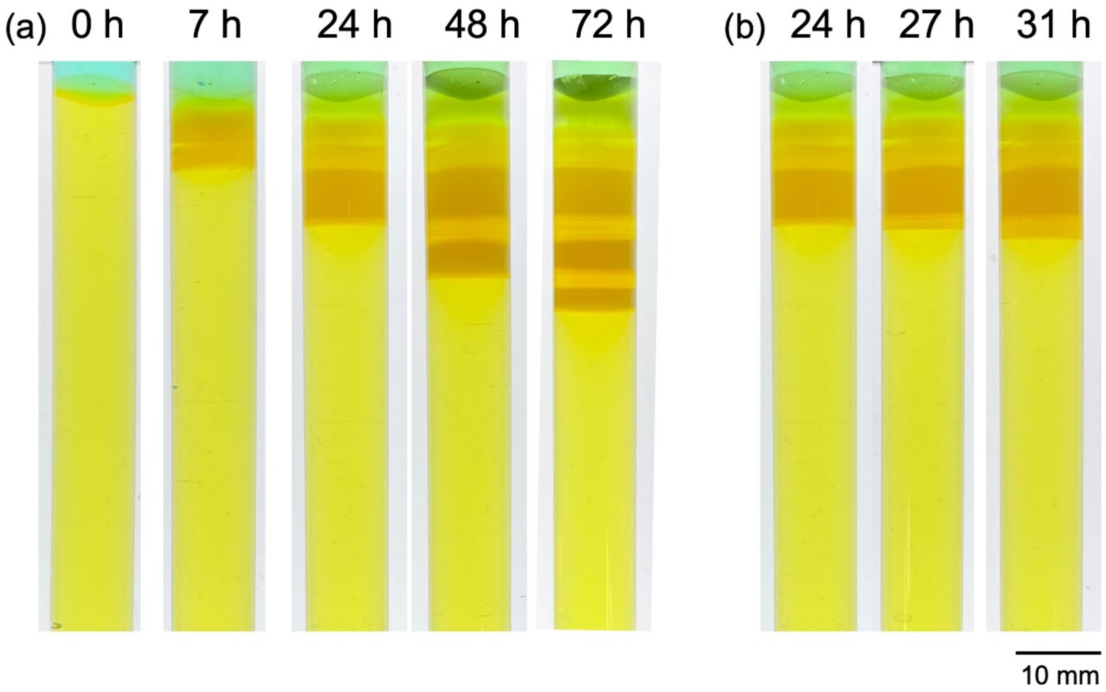

Figure S5. (a) Time course of pattern formation at φ_{DMSO} = 0.5 from 0 h to 72 h. (b) A detailed evolution of the formation of the inter-band region between two bands in the low-frequency revert pattern.

3.3 Pattern formation with other solvents (DMF, EG, TBA, GL, and GC)

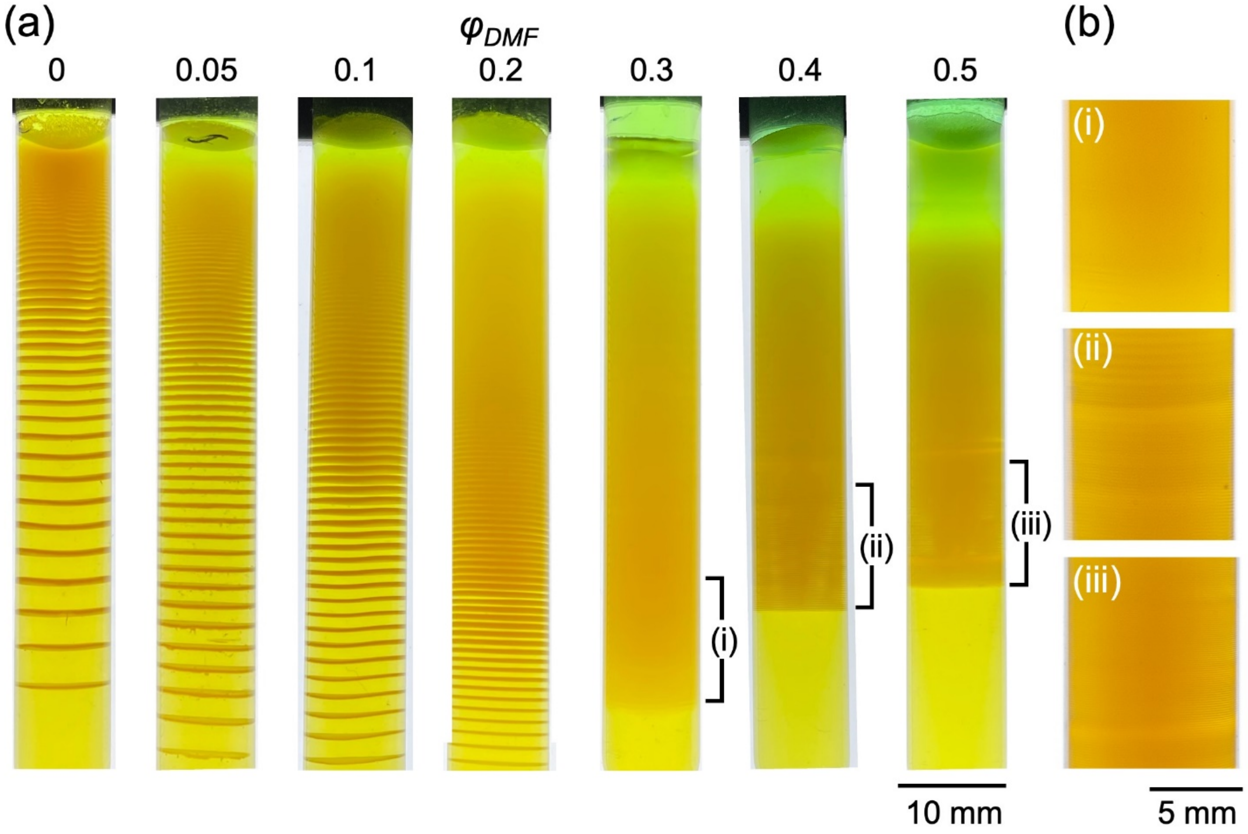

Figure S6. (a) Pattern formation of $CuCrO_4$ system with different volume ratios of DMF (φ_{DMF}). (b) Enlarged photographs of regions (i)–(iii) in (a).

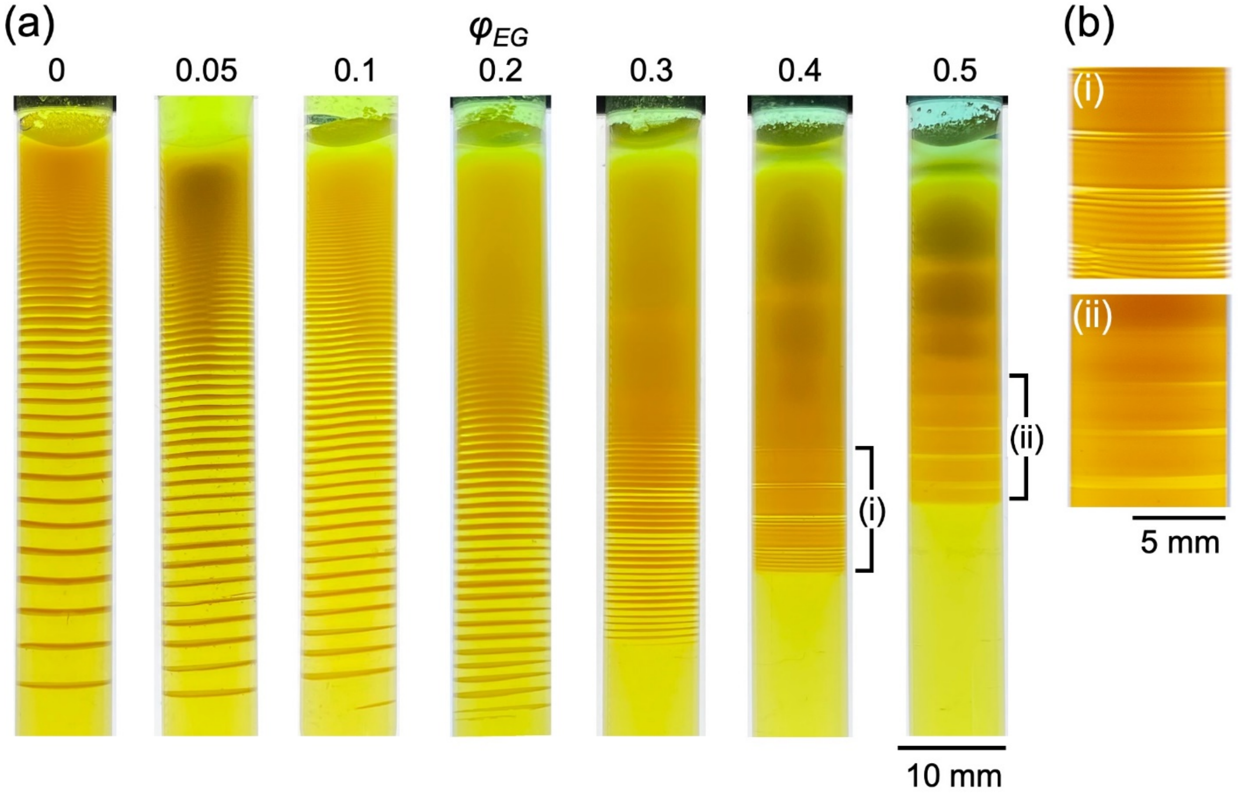

Figure S7. (a) Pattern formation of $CuCrO_4$ system with different volume ratios of EG (φ_{EG}). (b) Enlarged photographs of regions (i) and (ii) in (a).

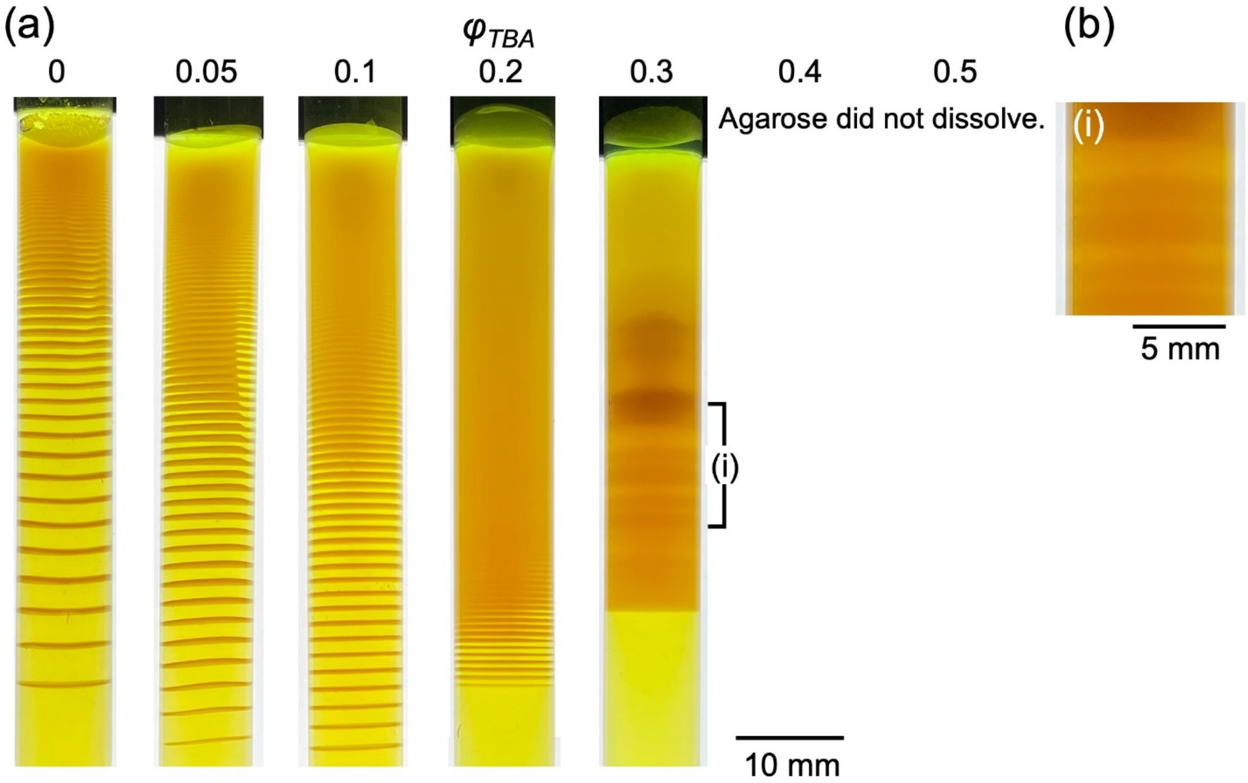

Figure S8. (a) Pattern formation of $CuCrO_4$ system with different volume ratios of TBA (φ_{TBA}). Gels with φ_{TBA} = 0.4 and 0.5 could not be prepared because the agarose did not dissolve. (b) Enlarged photographs of regions (i) in (a).

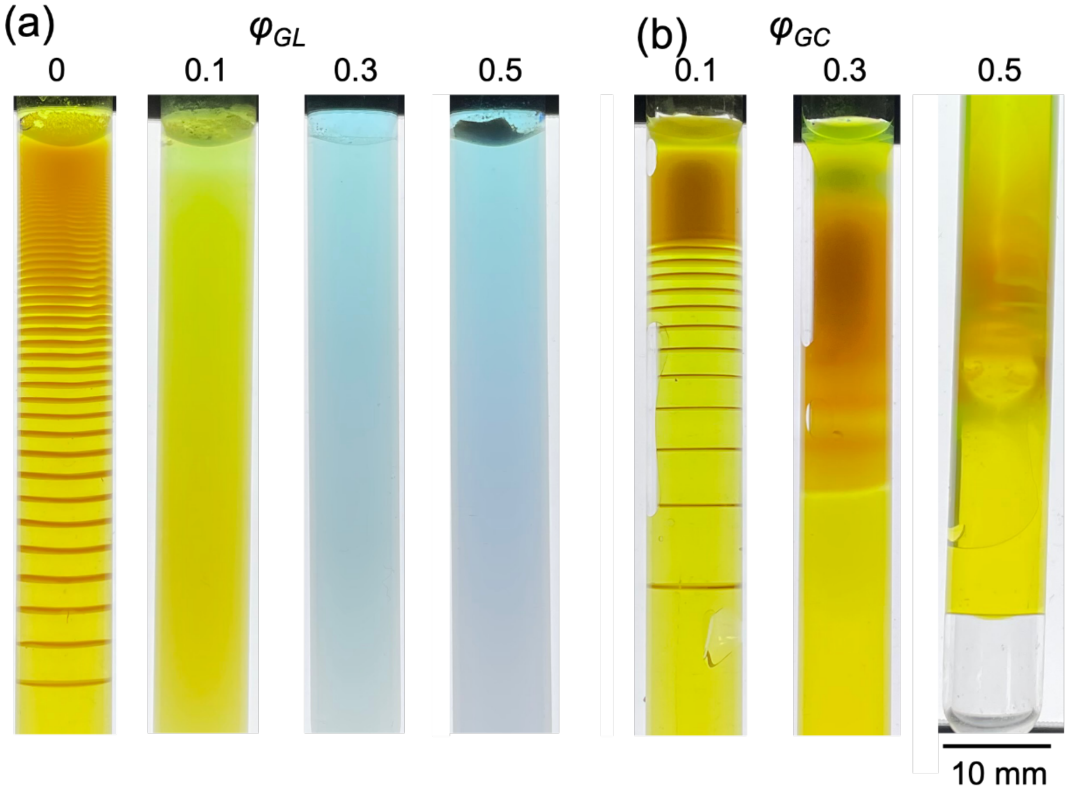

Figure S9. Pattern formation of $CuCrO_4$ system with different volume ratios of (a) GL (φ_{GL}) and (b) GC (φ_{GC}).

In the case of GL, the precipitate was not formed above $\varphi_{GL} = 0.1$. It is suggested that a redox reaction between CrO_4^{2-} and GL occurred because each substance is known as typical oxidative and reducing agent, respectively. Also, GC was produced by a reaction between GL and CO_2. Therefore, GC could have reacted with CrO_4^{2-} similarly to the case of GL. As evidence of this, voids were formed between the test tube and the gel due to the generation of gas and the gel was destroyed during the patterning process.

3.4 Width law for the revert-type LPs (low-frequency patterns)

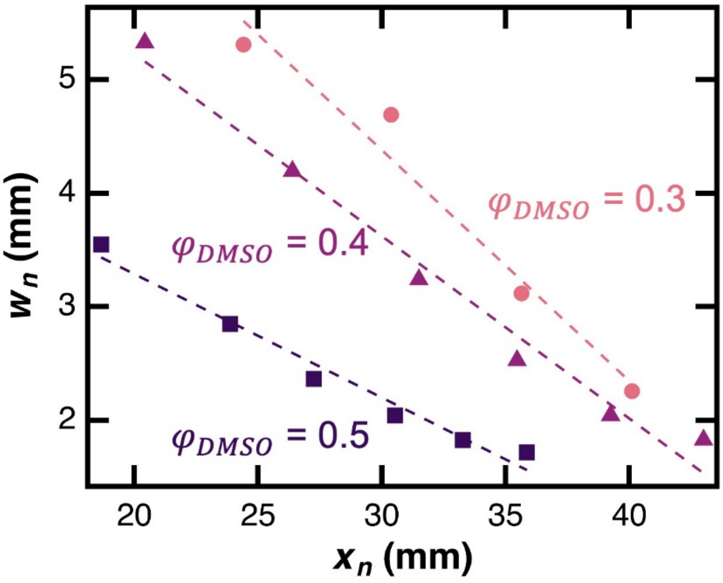

Figure S10. Variation of width of nth revert-type (low-frequency) band (w_n) against their position from the gel–solution interface (x_n) at φ_{DMSO} = 0.3 (●), 0.4 (▲), and 0.5 (■). All data were fitted by the linear function (R^2 = 0.964 (φ_{DMSO} = 0.3), 0.979 (φ_{DMSO} = 0.4), and 0.973 (φ_{DMSO} = 0.5)).

3.5 Line profile analysis

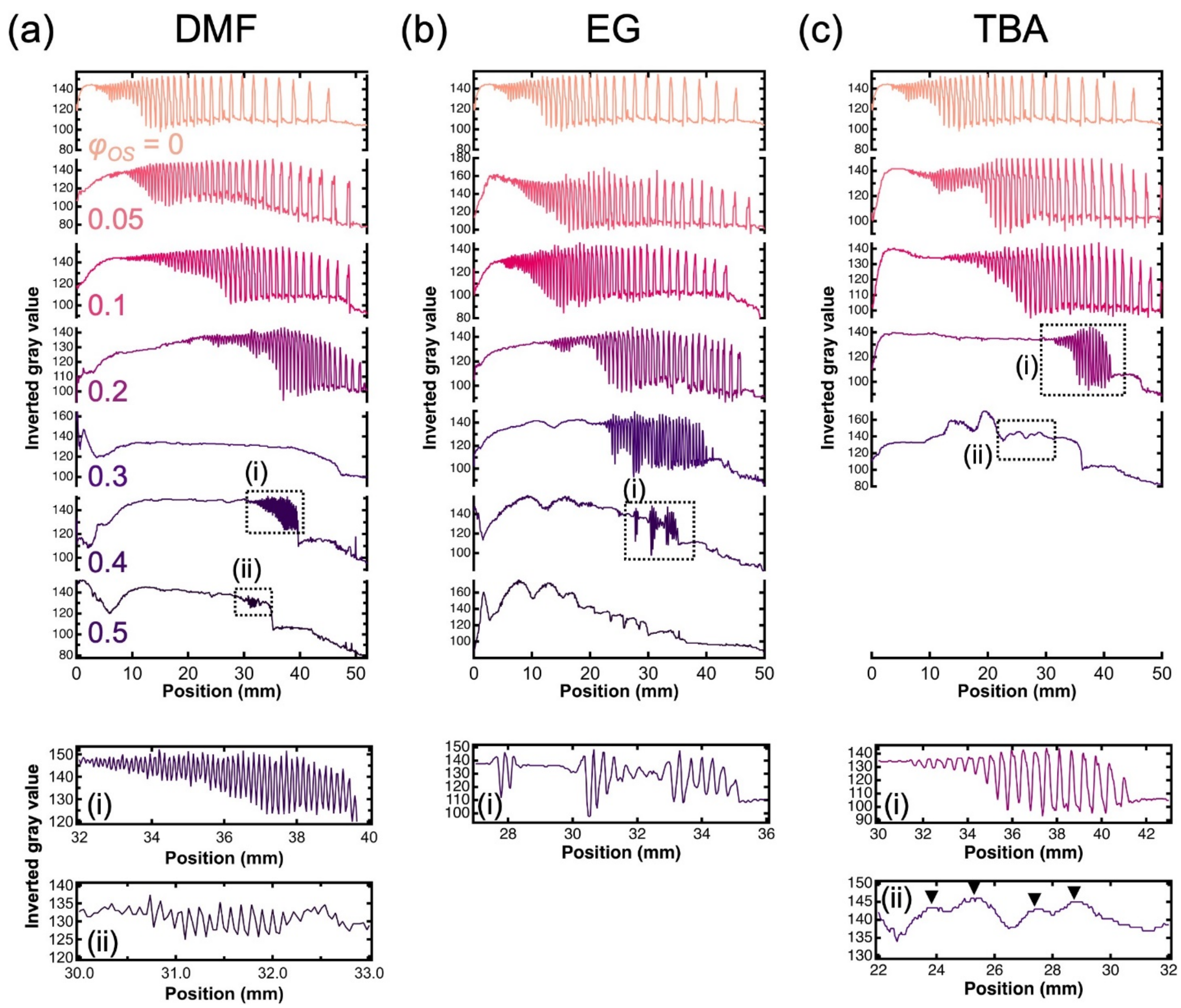

Figure S11. (a) Line profile analysis from the solution–gel interface (*position* = 0) to the bottom of test tubes with each φ_{OS} condition and enlarged figures for regions surrounded by doted square – (a): DMF, (b): EG, and (c) TBA. Triangle in the enlarged image (ii) with TBA shows splitting of bands.

3.6 Relationship between the spacing coefficient (*p*) and φ_{os}

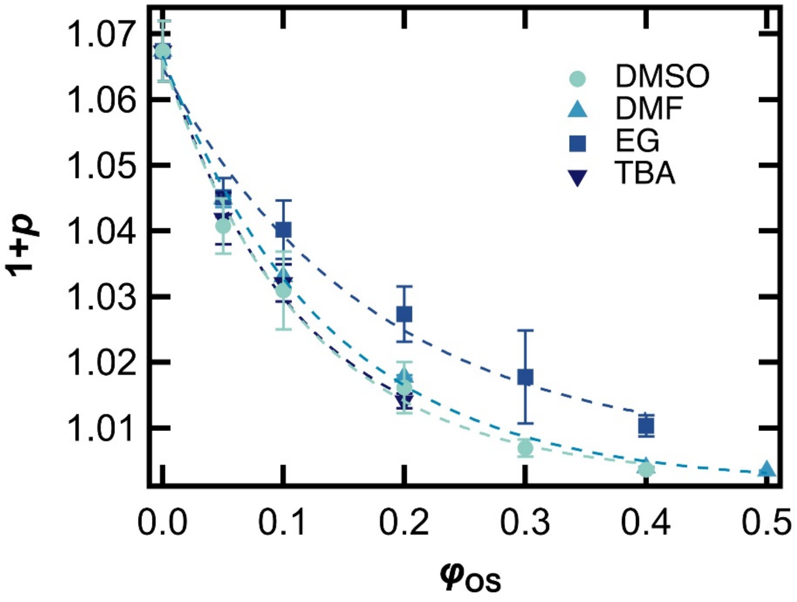

Figure S12. Relationship between the spacing coefficient (p) and φ_{OS} with DMSO (●), DMF (▲), EG (■), and TBA (▼)., p is calculated from the regular LP including the high-frequency pattern. The plot was fitted by a power function ($f(x) = f(0) + \alpha x^{\beta}$, where α and β are coefficients). Error bars were obtained from 5 times replications, which were calculated using p-values < 0.05.

3.7 DLS and zeta potential measurements

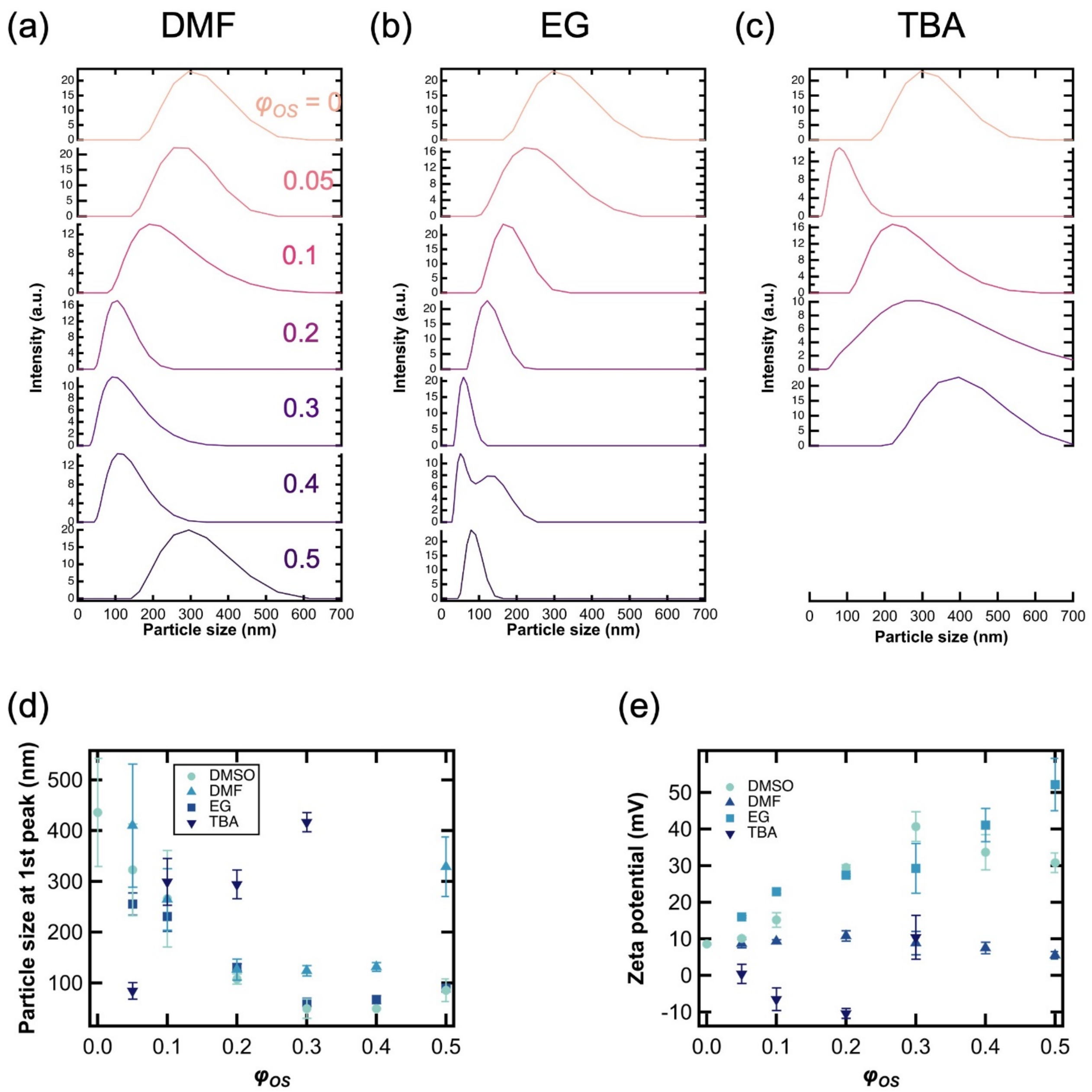

Figure S13. Size distribution of $CuCrO_4$ colloidal particles in aqueous solution with different φ_{OS} in cases of (a) DMF, (b) EG, and (c) TBA, measured by dynamic light scattering (DLS). (b) Relationship between mean particle size at 1st peak (with smaller size in binary distribution) and φ_{OS} (●: DMSO, ▲: DMF, ■: EG, and ▼: TBA) (c) Relationship between zeta potential of $CuCrO_4$ particles and φ_{OS}. Indicators represent the same conditions as (b).

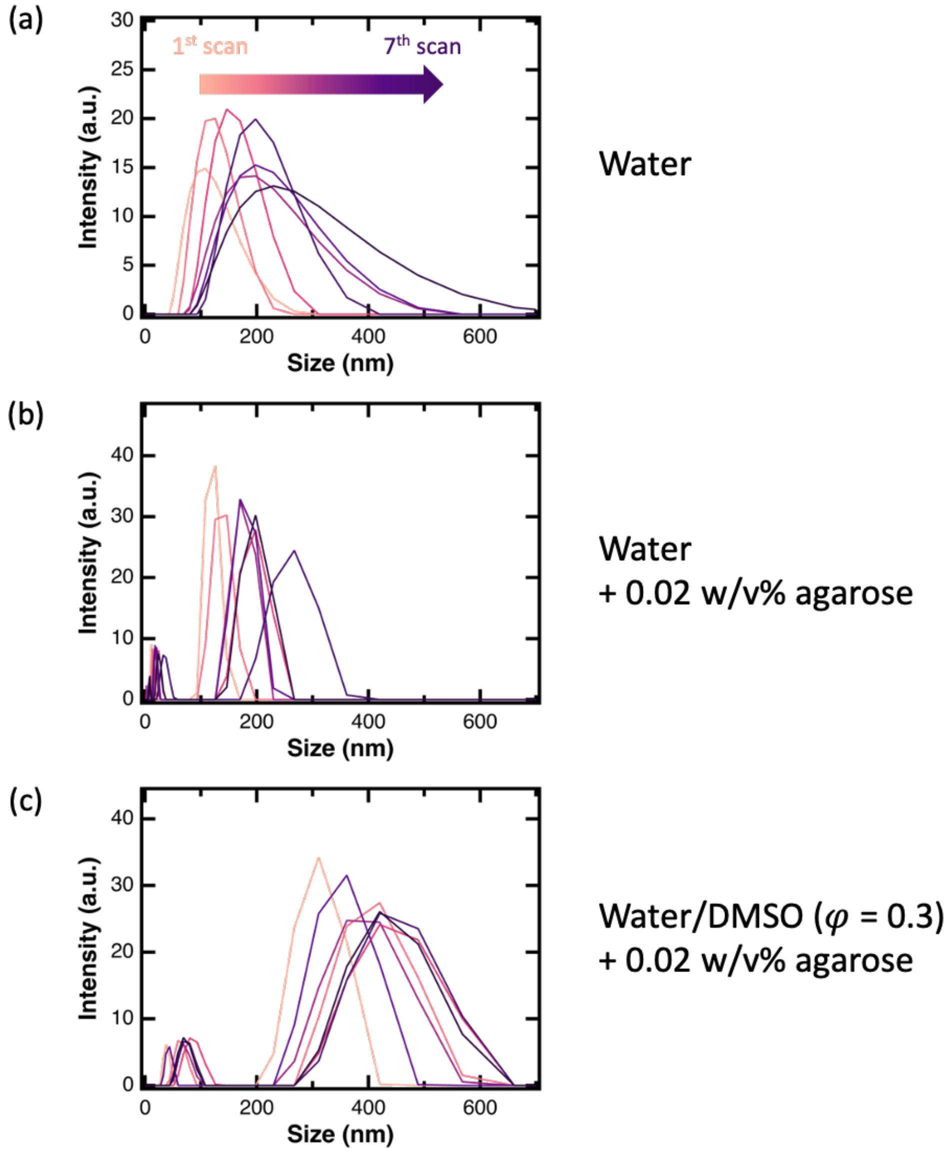

Figure S14. Kinetic measurement of $CuCrO_4$ particle size in solution with several conditions: (a) pure water, (b) 0.02 %w/v aqueous solution of agarose, and (c) water/DMSO mixture (φ_{DMSO} = 0.3) with 0.02 %w/v agarose. The color change from pink to purple corresponds to the number of the scans. The time interval between the 1st and 7nd scans was 10 min.

3.8 Effect of φ_{OS} on diffusion of Cu^{2+}

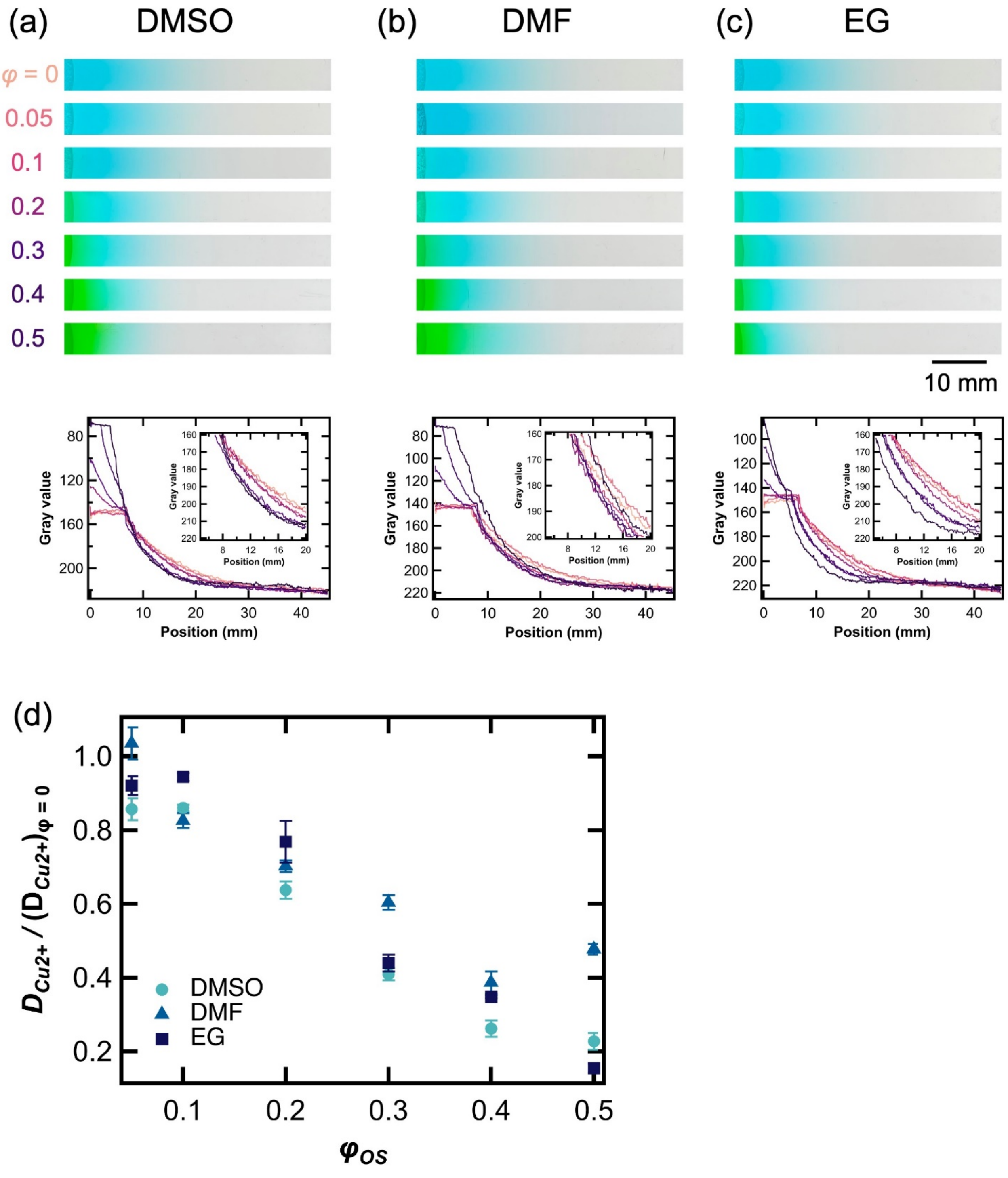

Figure S15. Photographs of simple diffusion experiment using Cu^{2+} after 1 day, and the line profile analysis with different φ_{OS} in cases of (a) DMSO, (b) DMF, and (c) EG. Experimental conditions were the following: [agarose] = 1.0 %w/v $[Cu^{2+}]_0$ = 4.0 M, and $[K_2CrO_4]$ = 0. Colors in the profile represent different values of φ_{OS}, and these correspond to colors of words denoted at the left side of images. (d) Ratio between diffusion coefficient of each φ_{OS} (D_{Cu2+}) and of φ_{OS} = 0 ($(D_{Cu2+})_{\varphi=0}$). These values were obtained by fitting the line profile with Fick's diffusion equation.

3.9 Simulations

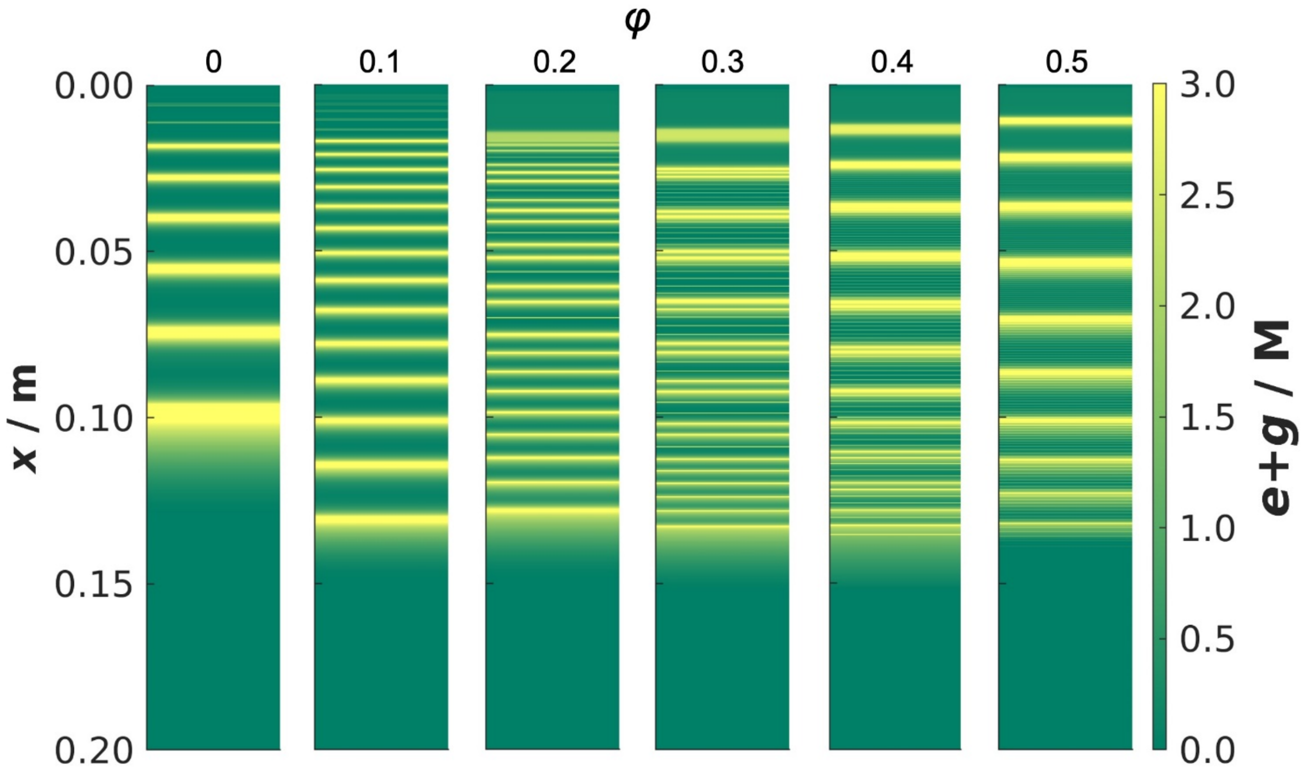

Figure S16. Resulted 2D images from numerical simulation with φ range from 0 to 0.5. Color changes from green to yellow shows sum concentration of E and G species ($e + g$), and value of concentration is indicated by color bar at the right side.

When φ increases from 0 to 0.1, inter-band spacing decreases, namely p decreases. As φ further increases to 0.2 and 0.3, low-frequency (thicker) and high-frequency (finer) patterns were formed. Finally, we can see the coexistence of the high and low-frequency patterns.

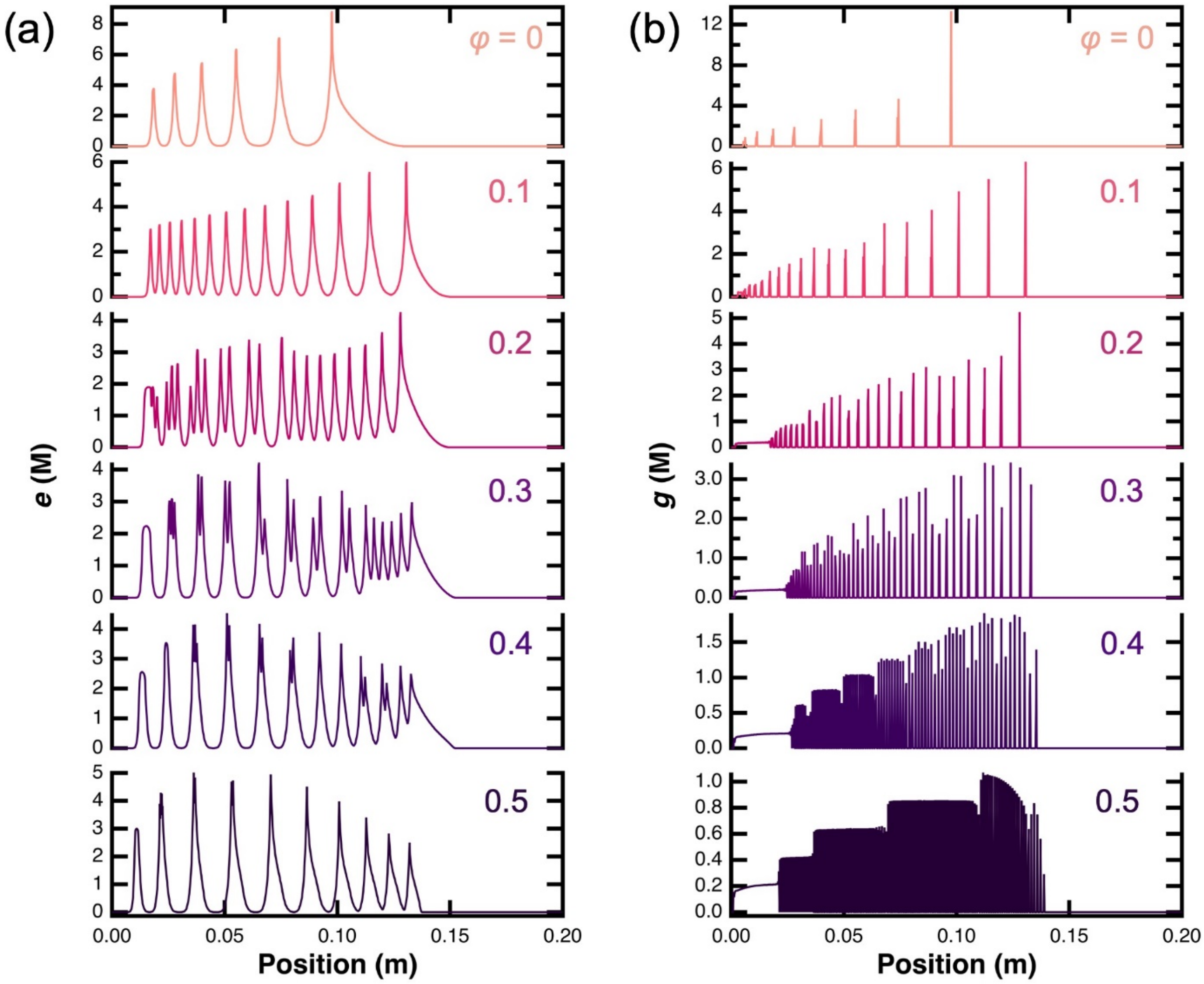

Figure S17. Concentration profiles in simulations at various φ: for the cases of (a) E in the homogeneous process and (b) G in the heterogeneous process.

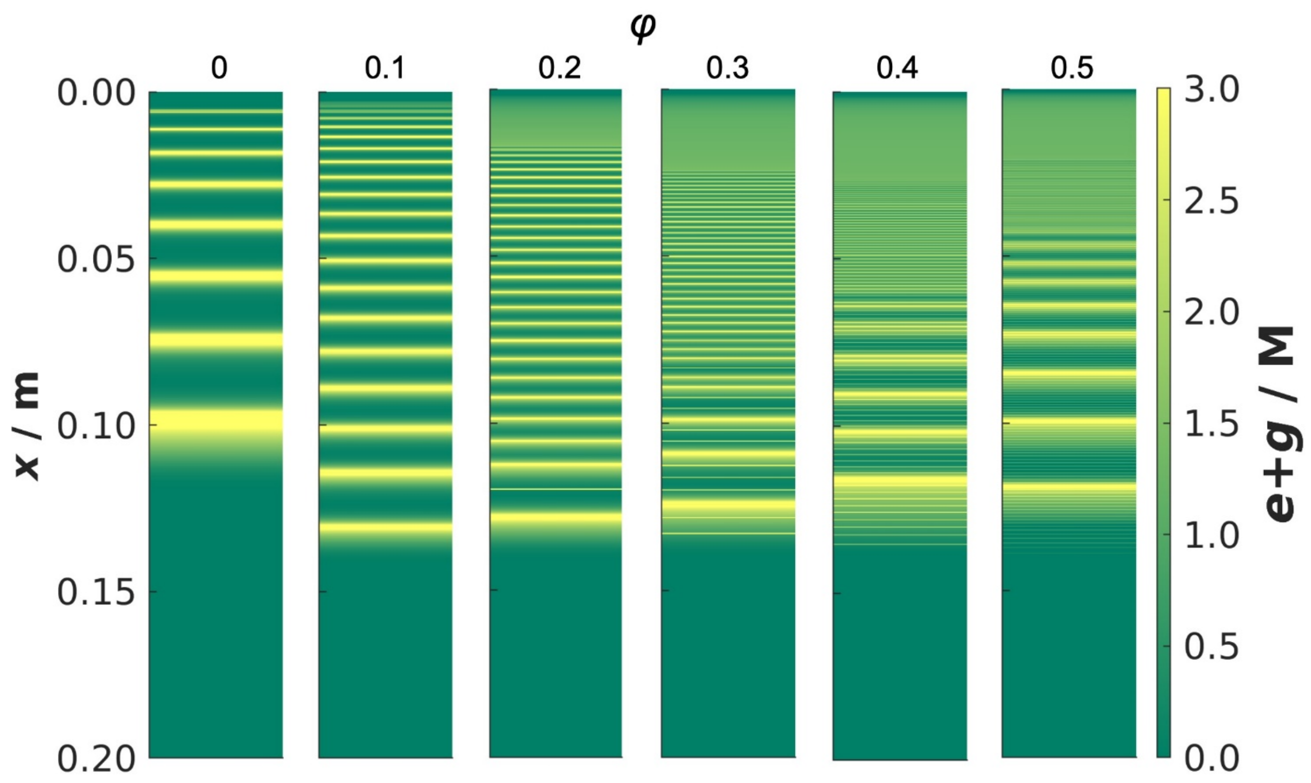

Figure S18. Results of the numerical simulations without the space dependence for D_C and c^*. The color change from green to yellow shows the sum concentration of E and G species (e + g), and the value of concentration is indicated by color bar at the right side.

To clarify the role of the spatial dependency on the D_C and c*, we carried out simulations using an alternative expression for these parameters by excluding the spatial dependency from the original equations (eqs. (19) and (21) in the SI). The used non-spatial dependence expression and parameter in this simulation are the following:

$$D_C = 0.55 \times D_A(\varphi) \;\; (\mathrm{m^2\,s^{-1}}), \qquad (22)$$

$$c^* = 0.4 \;\; (\mathrm{M}). \qquad (23)$$

It is noted that other parameters remained the same as we used in the original simulations. Difference from the original simulations with the spatial function is that the morphology of low-frequency patterns were the regular-type LPs even though φ increased up to 0.5. Therefore, we found that spatial dependency on these parameters is one of the essential factors to form the revert-type LPs.

3.10 K_{sp} measurements

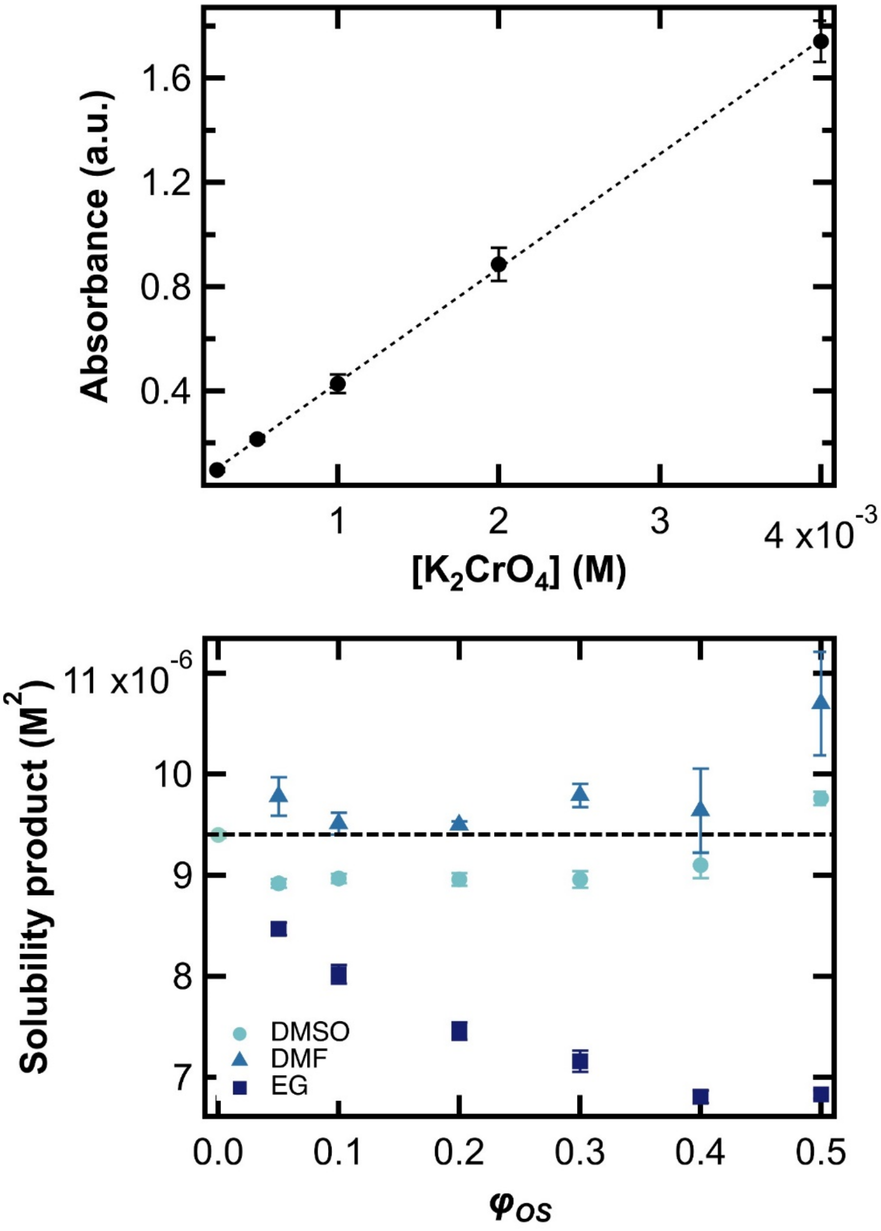

Figure S19. (a) Calibration curve as a function of K_2CrO_4 concentration. (b) Relationship between K_{sp} and φ_{OS}. The dashed line represents the value of K_{sp} when $\varphi_{OS} = 0$. In cases of DMSO (●) and DMF (▲), values of K_{sp} are almost same. However, K_{sp} decreases with increasing φ_{OS} in the case of EG (■).

4 References

1. Oh, K.-I.; Baiz, C. R. Crowding Stabilizes DMSO−Water Hydrogen-Bonding Interactions. *J. Phys. Chem. B* **2018,** *122*, 5984–5990.
2. Walliser, R. M.; Boudoire, F.; Orosz, E.; Tóth, R.; Braun, A.; Constable, E. C.; Rácz, Z.; Lagzi, I. Growth of Nanoparticles and Microparticles by Controlled Reaction-Diffusion Processes. *Langmuir* **2015,** *31*, 1828–1834.
3. Antal, T.; Droz, M.; Magnin, J.; Rácz, Z.; Zrinyi, M. Derivation of the Matalon-Packter law for Liesegang patterns. *J. Chem. Phys.* **1998,** *109*, 9479–9486.
